# Sigmar1’s Molecular, Cellular, and Biological Functions in Regulating Cellular Pathophysiology

**DOI:** 10.3389/fphys.2021.705575

**Published:** 2021-07-07

**Authors:** Richa Aishwarya, Chowdhury S. Abdullah, Mahboob Morshed, Naznin Sultana Remex, Md. Shenuarin Bhuiyan

**Affiliations:** ^1^Department of Molecular and Cellular Physiology, Louisiana State University Health Sciences Center-Shreveport, Shreveport, LA, United States; ^2^Department of Pathology and Translational Pathobiology, Louisiana State University Health Sciences Center-Shreveport, Shreveport, LA, United States

**Keywords:** Sigmar1, molecular structure, physiological function, cellular function, biological function

## Abstract

The Sigma 1 receptor (Sigmar1) is a ubiquitously expressed multifunctional inter-organelle signaling chaperone protein playing a diverse role in cellular survival. Recessive mutation in Sigmar1 have been identified as a causative gene for neuronal and neuromuscular disorder. Since the discovery over 40 years ago, Sigmar1 has been shown to contribute to numerous cellular functions, including ion channel regulation, protein quality control, endoplasmic reticulum-mitochondrial communication, lipid metabolism, mitochondrial function, autophagy activation, and involved in cellular survival. Alterations in Sigmar1’s subcellular localization, expression, and signaling has been implicated in the progression of a wide range of diseases, such as neurodegenerative diseases, ischemic brain injury, cardiovascular diseases, diabetic retinopathy, cancer, and drug addiction. The goal of this review is to summarize the current knowledge of Sigmar1 biology focusing the recent discoveries on Sigmar1’s molecular, cellular, pathophysiological, and biological functions.

## Introduction

### Sigma Receptor

Sigma receptors were first proposed to be a subclass of opioid receptors based on the observations of the psychotomimetic actions of (±)-SKF-10,047 (N-allylnormetazocine) and other racemic benzomorphans on behavior in dogs (Martin et al., 1976). The complex pharmacology of this racemic compound led to the naming of “Sigma opioid receptors” as a subtype of the opioid receptor family (Martin et al., 1976). Subsequent pharmacological and behavioral studies revealed (–)-SKF-10,047 binds to μ and κ opioid receptors, whereas the (+)-SKF-10,047 isomer binds with high affinity to the sigma receptor (Su, 1982). Therefore, the protein was named “Sigma receptor” by Su to distinguish it from opioid receptors (Su, 1982).

Two subtypes of Sigma receptors have been proposed based on their drug selectivity pattern and molecular mass: Sigma-1 receptor (Sigmar1) and Sigma-2 receptor (Sigmar2) (Su, 1982; Hellewell and Bowen, 1990). Sigmar1 is characterized by a higher affinity for dextrorotatory benzomorphans rather than its levorotatory isomers (Su, 1982). On the other hand, Sigmar2 exhibit an equal or greater affinity for the levorotatory benzomorphans isomers than their dextrorotatory counterparts (Hellewell and Bowen, 1990). Subsequent studies demonstrate that these two subtypes of Sigma receptors mediate different cellular and physiological functions. Though recent studies identified transmembrane protein 97 (TMEM97) as Sigmar2 (Alon et al., 2017), the literature contains conflicting evidence concerning the sequence, structure, and function of Sigmar2. Sigmar1 was successfully cloned in 1996 and has been more extensively examined in different research areas (Hanner et al., 1996). In this review article, we will focus on the recent discoveries concerning the molecular, cellular, pathophysiological, and biological functions of Sigmar1.

### Molecular Characterization and Structure of Sigmar1

Sigmar1 is a multifunctional, ubiquitously expressed chaperone protein encoded by the SIGMAR1 gene. The Online Mendelian Inheritance in Man (OMIM) catalog entry describes SIGMAR1 to be located in the p arm of Chromosome 9 with the cytogenetic location of 9p13.3 and genomic coordinates of 9:34,634,721–34,637,825 [according to the National Center for Biological Information (NCBI)].

#### Structure

The molecular characterization of Sigmar1 began with the purification and cloning of Sigmar1-binding site from guinea pig liver using Sigmar1 specific probes such as benzomorphan (+) [3H] pentazocine and arylazide(−)[3H] azidopamil (Hanner et al., 1996). The molecular mass as determined by radiation inactivation of a pentazocine-labeled Sigmar1-binding site yielded a value of 24 ± 2 kDa. However, subsequent cloning of cDNA using degenerate oligonucleotides and cDNA library screening showed Sigmar1 protein isolated from guinea pig liver had 223 amino acids (aa) with a molecular mass of 25,314 Da (25.3 kDa) with at least one putative transmembrane segment. Human Sigmar1 (hSigmar1) cloned from human placental choriocarcinoma (JAR) cells cDNA library also predicted to have a protein of 223 amino acids with a single putative transmembrane domain (Kekuda et al., 1996). Sigmar1 mRNA (1.7 kb) was expressed in several human and guinea pig tissues, and the highest densities were found in liver, kidney, and steroid producing tissues such as placenta, ovary, and adrenal gland (Hanner et al., 1996; Kekuda et al., 1996). Subsequent cloning and functional characterization of mouse and rat Sigmar1 showed similar results having 223 aa (Seth et al., 1997, 1998; Mei and Pasternak, 2001). The sequence of murine Sigmar1 showed homology to guinea pigs (87% identity and 91% similarity), rats (92% identity and 96% similarity), and humans (90% identity and 93% similarity) (Seth et al., 1997). Rat Sigmar1 has an open reading frame of 672 base pairs (bp) flanked with non-coding regions of 30 bp at 5′ and 880 bp at 3′ of the coding region (Seth et al., 1998). Rat Sigmar1 has two transmembrane domains with 93.3% sequence homology with the mouse Sigmar1, 93.7% with that of guinea pig, and 96.0% with that from human (Mei and Pasternak, 2001).

**FIGURE 1:**
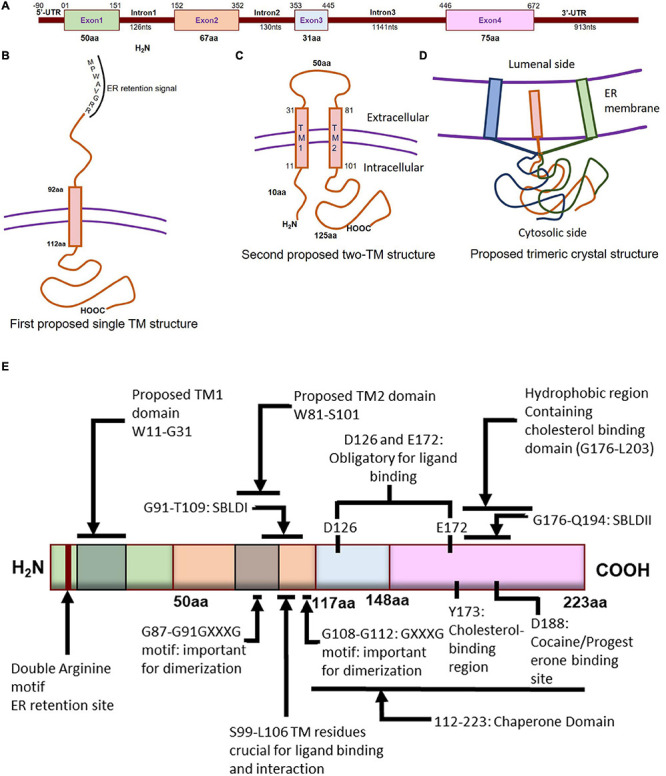
Molecular characterization of Sigmar1. **(A)** Schematic diagram representing the genetic structure of the SIGMAR1 gene, including the exon and, intron lengths in terms of nucleotides and amino acids. **(B)** Simplified schematic of full-length Sigmar1 topology showing the first predicted one transmembrane (TM) structure with a TM domain-containing amino acid (aa) residues from 92 to 112, MPWAVGRR as the ER retention site, and regions important for ligand binding (exon 3 and Ser99-Leu106). **(C)** Simplified schematic of the full-length Sigmar1 topology showing the second predicted two TM structures with two TM domains (TM1 containing aa residues 11 to 31 and TM2 containing 81 to 101) and an extracellular loop (containing 50aa). Both N- and C- terminal of the protein are on the same side. **(D)** Simplified schematic for the recent crystal structure of full-length Sigmar1 suggesting the trimeric structure of the protein with trimerization of three single transmembrane domains and each transmembrane domain being tightly associated with one promoter. The C-terminal is located on the cytosolic side. **(E)** Schematic diagram representing full protein structure of Sigmar1 with structural details for receptor dimerization, ligand binding, cholesterol-, cocaine-, progesterone- binding.

Both the murine and human Sigmar1 gene (approximately 7 kb) is made up of 4 exons and 3 introns: exon 3 is the shortest one, and exon 4 of the protein is the longest one (Seth et al., 1997; Prasad et al., 1998; Figure 1A). Both mouse and rat Sigmar1 cDNA has a poly(A) tail, an upstream polyadenylation signal (AATAAA), and the protein has an amino acid sequence of MPWAVGRR at the N-terminal (believed to be ER retention signal) (Seth et al., 1997, 1998). Subsequent studies have identified the presence of a Phenyl-A region in Sigmar1 as a crucial structural feature in determining the substrate specificity for Sigmar1 ligands (Ablordeppey et al., 2002). Furthermore, the structural analysis showed that the two arginine motifs at the N-terminus of Sigmar1 are required for ER membrane targeting (Schutze et al., 1994). In addition, studies have shown that the presence of Ser99 to Leu106 residues in Sigmar1 protein located in the putative transmembrane domain play a crucial role in ligand binding and receptor-ligand interaction (Yamamoto et al., 1999). Investigation of Sigmar1 ligand binding sites in Jurkat human T lymphocyte suggested two spliced variants lacking exon 3 (deletion of 31 amino acids), which failed to bind with Sigmar1 ligands suggesting Exon 3 as the ligand-binding (Ganapathy et al., 1999). Subsequent studies identified six spliced variants in mouse Sigmar1 formed either by exon skipping or alternative 3′ and 5′ splicing to generate truncated proteins. Differential expressions of these Sigmar1 variants were observed across different organs (Pan et al., 2017).

#### Transmembrane Topology

Guinea pig Sigmar1 showed substantial sequence homology with fungal sterol C8-C7 isomerase (ERG2: 30.3% identical and 66.4 similarity), and hydrophobicity plots predicted one putative transmembrane segment at the N terminus (Hanner et al., 1996; Figure 1B). However, unlike the fungal sterol C8-C7 isomerase (Hanner et al., 1996), the Sigmar1 lacks sterol isomerase activity and shares no sequence homology with any known mammalian proteins, including the mammalian C8-C7 sterol isomerase (Labit-Le Bouteiller et al., 1998). Further, studies from two independent groups (Aydar et al., 2002; Hayashi and Su, 2007) suggested Sigmar1 having two transmembrane domains where both N- and C- terminal of Sigmar1 resides on the same side of the membrane. However, the proposed model by Aydar et al. suggested that both the N- and C-termini being intracellular, whereas the proposed model by Hayashi et al. showed them extracellular. Aydar et al. further proposed transmembrane domain 1 with amino acid (aa) residues 9 to 28 and transmembrane domain 2 with residues 81–101 (Aydar et al., 2002). Studies also suggested that Sigmar1 has a short N-terminal (10 aa), an extracellular loop (50 aa), and a longer C-terminal (125 aa) with a sequence similar to sterol isomerase (Aydar et al., 2002). Moreover, Sigmar1’s both N- and C- terminal are hypothesized to reside intracellularly (Aydar et al., 2002; Figure 1C).

More recently, Sigmar1’s crystal structure determined by overexpressing the FLAG-tagged Sigmar1 in baculovirus, affinity purification and reconstitution of the protein into lipidic cubic phase, and crystallization by the hanging drop technique (Schmidt et al., 2016). The crystal structure of the Sigmar1 protein was suggested to possess a single TM domain with a short N-terminus facing the ER lumen, while most of the protein bulk was located on the cytosolic side of the ER membrane (Schmidt et al., 2016; Figure 1D). In contrast, Sigmar1 transmembrane topology determined by electron microscopic examination of ascorbate peroxidase 2 (APEX2)-tagged Sigmar1 protein in transfected ND7/23 cells suggested the N-terminus of Sigmar1 facing the cytosol and the C-terminus facing the ER lumen (Mavylutov et al., 2018). Though the Sigmar1’s topology identified in the GFP-APEX2-tagged Sigmar1 and the crystal structure study (Schmidt et al., 2016) showed similarity, they differ whether the facing of the N-terminus of Sigmar1 protein faces the cytosol or ER lumen.

#### Ligand Binding

Previous work using site-directed mutagenesis showed Asp126 and Glu172 are essential for high-affinity ligand binding, as mutation of either resulted in a profound loss of ligand-binding activity (Seth et al., 2001; Figure 1E). Study using the hydropathy plot suggested that a hydrophobic segment (aa 176 to 203) in Sigmar1 contains the cholesterol-binding domain with the conserved L/V-X1-5- Y-X1-5-K/R motif near the ligand-binding domain (Palmer et al., 2007). This study further suggested that amino acid residue Y173 is crucial for cholesterol binding (Palmer et al., 2007). Later studies involving photolabeling of the protein have suggested that Sigmar1 has two sterol binding domain-like motifs, namely SBLDI (aa 91–109) and SBLD II (aa 176–194). Further studies have demonstrated that these two domains are close enough and juxtaposed to form a ligand-binding site responsible for ligand binding and lipid raft remodeling (Fontanilla et al., 2008; Pal et al., 2008). 3D modeling of the Sigmar1 protein structure supports the above-described study, providing further evidence for the presence of β-strands in the C-terminal half of the protein (at residues 111–116, 133–135, 144–146, and 158–164) (Laurini et al., 2011). The crystal structure of Sigmar1 also suggested the Sigmar1 ligand binding through a charge-charge interaction with the highly conserved Glu172 and Asp126 (Schmidt et al., 2016).

#### Oligomerization

Several studies suggested the existence of the oliogomeric structure of Sigmar1 and ligand-dependent changes in Sigmar1 oligomerization as well as activity. Studies using photo-affinity labeling have demonstrated the dimeric structure or oligomer of dimeric structures of Sigmar1, which is associated with the presence of two GXXXG motifs at residues 87–91 (TM2) and residues 108–112 (C-terminal of SBLDII) (probable oligomerization motifs) (Chu et al., 2013). Fluorescence resonance energy transfer spectrometry analysis of heterologously expressed Sigmar1 in COS-7 cells showed the presence of multiple oligomeric forms. Treatment with Sigmar1 ligands altered these oligomer forms where Sigmar1 agonist [(+)-pentazocine] favored the monomers and dimers, and Sigmar1 antagonist (haloperidol) favored higher order Sigmar1 oligomers (Mishra et al., 2015). The crystal structure of Sigmar1 proposed a trimeric structure of Sigmar1 where Sigmar1 possesses a single transmembrane domain at N-terminus (Schmidt et al., 2016). Size-exclusion chromatography with multi-angle light scattering experiments as well as native polyacrylamide gel electrophoresis analysis suggested the presence of Sigmar1 oligomers ranging in size from hexamers to as large as 15-mers (Schmidt et al., 2016). All these studies suggested ligand mediated oligomerization as an important characteristics for Sigmar1 activity, but the molecular mechanism of Sigmar1 oligomerization and resultant changes in Sigmar1’s function remained elusive.

### Tissue Distribution of Sigmar1

Extensive Northern blot assays carried out in animals and humans have demonstrated the ubiquitous expression of Sigmar1 throughout the body tissures, including heart, liver, brain, placenta, thymus, lung, kidney, stomach, skeletal muscle, and pancreas (Kekuda et al., 1996; Mei and Pasternak, 2001). The characterization of spliced variants in mice revealed the presence of full-length protein and all of the spliced variants of Sigmar1 across different organs, including lung, liver, heart, spleen, kidney, brain, and various regions (Pan et al., 2017). Sigmar1 has been shown to be expressed in the spleen in mice and guinea pigs (Su et al., 1988; Mei and Pasternak, 2001) and in peripheral blood leukocytes in humans (Wolfe et al., 1988). However, expression of Sigmar1 protein levels in different tissues varied, with the highest expression in the liver (Kekuda et al., 1996; Mei and Pasternak, 2001; Pan et al., 2017). The Human Protein Atlas (^1^ /ENSG00000147955 -SIGMAR1/tissue) summarizes the expression of Sigmar1 across different tissues of the human body and shows the highest level of Sigmar1 expression in the brain (cerebellum), liver and placenta; moderate levels in heart, skeletal muscle, different glands (parathyroid, adrenal, thyroid), pancreas, lungs, GI tract, kidneys, urinary bladder, and male and female reproductive organs; and low levels in soft tissue, with no report of expression in the bone marrow. Studies on Sigmar1 expression at the sub-tissue level have shown its presence in astrocytes, oligodendrocytes, gangliosides, and basal amygdala of the neuronal system (Palacios et al., 2003; Choi et al., 2016; Kasahara et al., 2017; Zhang et al., 2017a). Moreover, Sigmar1 also has been found in retinal tissue, bile duct, breast tissue, bone marrow-derived macrophages, endothelial cells (Amer et al., 2013; Barbieri et al., 2003; Xu et al., 2014; Mavlyutov et al., 2015a; Rosen et al., 2019). Despite its ubiquitous tissue distribution, studies to date have only attempted to explore the pathophysiological role of Sigmar1 in the neuronal, cardiovascular, kidney, and retinal systems.

### Subcellular Localization

**FIGURE 2:**
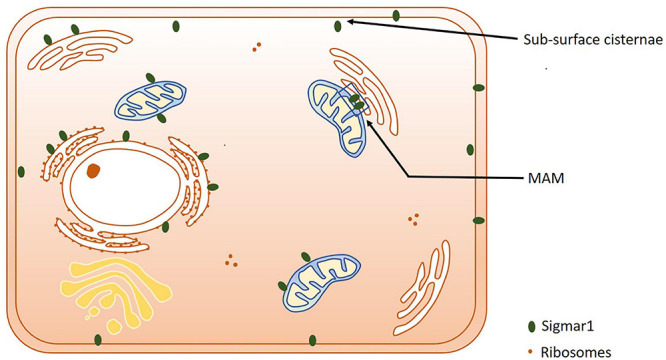
Subcellular localization of Sigmar1. Cartoon showing a summary of the sub-cellular localization of Sigmar1 as evidenced by several studies using various cell types. Overall, the presence of Sigmar1 has been detected on the mitochondria-associated ER membrane (MAM), plasma membrane, ER membrane, nuclear membrane, mitochondria-associated ER membrane, mitochondrial membrane, nucleoplasmic reticulum and sub-surface cisternae in different cell types including CHO cells, human B and T cells, photoreceptor cells, and neuronal cell lines including NSC34 cells and neuro2a cells.

Extensive studies over the last 40 years have demonstrated that the subcellular localization of Sigmar1 is tissue-specific. Comprehensive studies have shown Sigmar1 localization at the mitochondrial-associated membranes (MAM) (co-localized with Mito-DsRed) in CHO Cells (Hayashi and Su, 2007) and the plasma membrane, where it interacts with ion channels (reviewed in Su et al., 2009). Studies have also shown localization of Sigmar1 at the endoplasmic reticulum (ER) and nuclear envelope in human immune cells (Dussossoy et al., 1999). Sigmar1 present in the cell membrane negatively regulating Kv1.4 potassium channel function (Aydar et al., 2002). Extensive immuno-electron microscopic (EM) data have shown that Sigmar1’s sub-cellular localization largely depends on cell and organ types (Mavlyutov and Ruoho, 2007; Mavlyutov et al., 2010, 2011, 2012, 2013, 2015a, 2016, 2017a; Mavlyutov and Guo, 2017; Yang et al., 2017). For example, Sigmar1 was localized to the nuclear envelope with no localization was observed in ER in the photoreceptor cells (Mavlyutov et al., 2015a), whereas Sigmar1 localization was observed in the nucleoplasmic reticulum and the nucleus in the NSC34 cell line (Mavlyutov et al., 2017; Figure 2 and Table 1). Moreover, immuno-EM examinations were unable to detect Sigmar1 at the plasma membrane (Mavlyutov and Ruoho, 2007; Mavlyutov et al., 2010, 2011, 2012, 2013, 2015a, 2016, 2017a; Mavlyutov and Guo, 2017; Yang et al., 2017). Mavlyutov group also showed that Sigmar1’s C-terminal resides inside ER-lumen and the N-terminus resides in the cytosol (Mavylutov et al., 2018), which is opposite to the recently derived crystal structure proposing that Sigmar1’s C-terminal reside on the cytosolic side of the ER (Schmidt et al., 2016). Sigmar1 was also detected on mitochondria of rat liver (depicted as Sigmar1-like receptor) using ligand-based studies and immunostaining (Klouz et al., 2002). Using ligand binding assays using (+) pentazocine and enzyme binding or activity assays (monoamine oxidases, cytochrome c oxidases) in the mitochondrial fraction from rat liver and brain, the group show Sigmar1 to reside in the outer mitochondrial membrane and this Sigmar1 in the liver to have a different binding site for ligands compared to that in the brain (Klouz et al., 2002). The existence of Sigmar1 on the mitochondria was confirmed by colocalization of Sigmar1 on mitochondria on liver tissue section when stained with Sigmar1 antibody and a mitochondrial marker (Klouz et al., 2002). Interestingly, subcellular fractionation of neural tissues from the mutant SOD1Tg mice showed Sigmar1 accumulation in mitochondrial fractions (Watanabe et al., 2016). The apparent discrepancies’ in Sigmar1’s subcellular localization results from the differences in cell types, methods of detection, and reagents used (i.e., antibody) (summarized in Table 1). All these studies to date suggest the organ- and tissue-specific localization and function of Sigmar1 (Mavlyutov and Ruoho, 2007; Mavlyutov et al., 2010, 2011, 2012, 2013, 2015a, 2016, 2017a; Mavlyutov and Guo, 2017; Yang et al., 2017).

**TABLE 1:** Major studies on identifying Sigmar1’s subcellular organelle localization (in chronological order).

**Subcellular organelles**	**Experimental methods**	**Cells or Tissues**	**Antibody and antigen**	**Antibody validation**	**References**
Mitochondria Microsomal Synaptosomal fractions	Western blotting on subcellular fractions	Rat brain	Anti-Sigmar1 Rabbit Polyclonal antibody raised against synthetic amino acid residues 143–163 of Sigmar1 (produced by authors)	Not listed	Yamamoto et al., 1999
Plasma membrane Mitochondrial membranes Vesicles or elongated cisternae of ER	Immunoelectron microscopy	Adult rat brain hypothalamus and hippocampus sections	Anti-Sigmar1 Rabbit Polyclonal IgG raised against synthetic peptide of Sigmar1 amino acid sequence 143–162 (produced by authors)	Antibody specificity confirmed through absence of staining in brain tissue sections when co-incubated with Sigmar1 specific antigen	Alonso et al., 2000
Perinuclear areas Plasmalemmal regions of cell-cell contact Growth cones of neurites	Fluorescence microscopy of co-immunocytohistochemistry and co-immunoprecipitation of endogenous Sigmar1 with Ankyrin B and IP3R3 receptors	Mouse neuroblastoma x rat glioma hybrid NG-108-15 cells	Anti-Sigmar1 Rabbit antibody raised against N-terminal residues of mouse Sigmar1 (produced by authors)	Not listed	Hayashi and Su, 2001
Mitochondrial membranes	i. Radioisotope labeled Sigmar1 agonist [^3^H](+)-pentazocine binding assay ii. Immunofluorescence	i. Isolated rat liver mitochondria ii. Rat liver sections	Anti-Sigmar1 Rabbit Polyclonal IgG (Alonso et al., 2000)	Validated by original contributors at (Alonso et al., 2000)	Klouz et al., 2003
Plasma membranes	i. Exogenous expression of N-terminal and C-terminal GFP tagged Sigmar1 constructs and fluorescence microscopy ii. Co-immunoprecipitation with anti-Kv1.4 antibody	i. *Xenopus* Oocytes ii. Triton X-100 solubilized membrane lysates of rat pituitary gland	Anti-Sigmar1 Rabbit Polyclonal IgG (Yamamoto et al., 1999)	Not listed	Aydar et al., 2002
i. ER associated detergent insoluble lipid droplets/microdomains ii. Sigmar1 immunostained reticular structure was noted negative for endosomes (EEA-1), mitochondria (Mitotracker, bcl-2), lysosomes (Lyso Tracker, LAMP-1), synaptic vesicles (Synapsin II), plasma membrane (Fas and CTx-B), golgi (GM130), ER-associated proteins	Fluorescence microscopy; cells were specially treated with 0.02% SDS for 10 min	NG-108 cells (Hayashi and Su, 2003a, b), Rat hippocampal differentiated oligodendrocytes culture (Hayashi and Su, 2004)	i. Exogenously expressed C-terminal Enhanced Yellow Fluorescent Protein (EYFP)-tagged mouse Sigmar1 construct ii. Polyclonal rabbit anti-guinea pig Sigmar1 antibody raised against guinea pig Sigmar1 amino acid sequence 144–165 (Hayashi and Su, 2003b).	i. Exogenous overexpression ii. Anti-Sigmar1 staining pattern have been visually compared to expression pattern of exogenously expressed Sigmar1-EYFP plasmid construct (Hayashi and Su, 2003a, b)	Hayashi and Su, 2003a, b, 2004
Mitochondria Mitochondria-associated ER membranes (MAM) Nuclear and microsomal fractions	i. Immunofluorescence staining of endogenous Sigmar1 in cells transfected with Mitochondria and ER fluorescent constructs, ii. Western blot analysis on ultracentrifugation based subcellular fractionations	Chinese hamster ovary (CHO) cells	Anti-Sigmar1 Rabbit antibodies raised against amino acid residues 52–69 and 143–165 of Rat Sigmar1 (produced by authors)	Western blot on CHO cell lysates and on different rat organs	Hayashi and Su, 2007
Focal adhesion contacts (FACs)	Co-localization of Sigmar1 with FAC protein Talin in immunofluorescence staining	CHO-K1 cells	Anti-Sigmar1 Rabbit antibody raised against Maltose Binding Protein (MBP)-Guinea Pig full length Sigmar1 fusion protein (Ramachandran et al., 2007) (produced by authors)	Western blot analysis using guinea pig Sigmar1 overexpressing COS-7 cells as positive control (Ramachandran et al., 2007)	Mavlyutov and Ruoho, 2007
i. Motor neurons of mouse brain Medulla and spinal cord ii. Cholinergic post-synaptic terminals co-localized to Kv2.1 potassium channels iii. Putative subsurface ER cisternae close to plasma membrane	i. Immunohistochemistry ii. Co-immunofluorescence with cholinergic neuron markers iii. Immuno-electron microscopy	Mouse brain and spinal cord sections	Anti-Sigmar1 Rabbit antibody raised against Maltose Binding Protein (MBP)-Guinea Pig full length Sigmar1 fusion protein (Ramachandran et al., 2007)	Sigmar1 knockout mice brain sections as negative control in immunohistochemistry	Mavlyutov et al., 2010, 2012
Mitochondria ER	Fluorescence microscopy	Mouse neuroblastoma Neuro-2a cells	Exogenously expressed eGFP and mCherry tagged Sigmar1’s correlative, visual colocalization observation with erRFP and mtGFP labeled ER and mitochondria, respectively	Exogenous overexpression	Shioda et al., 2012
Mitochondria and MAM fractions	Western blot analysis on subcellular fractions	Chinese hamster ovary (CHO) cells	Anti-Sigmar1 Rabbit antibodies raised against Rat Sigmar1 (Hayashi and Su, 2007)	Western blot on CHO cell lysates and on different rat organs (Hayashi and Su, 2007)	Mori et al., 2013
ER and Nuclear Envelope	Confocal immunofluorescence staining	NG108 and Neuro-2a cells	Anti-Sigmar1 (Santa Cruz Biotechnology)	Commercial vendor	Tsai et al., 2015
Predominantly in nuclear envelope, sparsely at ER cisternae located subsurface of plasma membranes	Immuno-electron microscopy	Mouse retinal photoreceptor cells	Anti-Sigmar1 Rabbit antibody raised against Maltose Binding Protein (MBP)-Guinea Pig full length Sigmar1 fusion protein (Ramachandran et al., 2007)	Reported at (Ramachandran et al., 2007)	Mavlyutov et al., 2015b
Plasma membrane ER Nuclear envelope	Immuno-electron microscopy	Mouse and rat dorsal root ganglion (DRG) cells	Anti-Sigmar1 Rabbit antibody raised against Maltose Binding Protein (MBP)-Guinea Pig full length Sigmar1 fusion protein (Ramachandran et al., 2007)	Validated through absence of immunofluorescence staining using Sigmar1 knockout mouse DRG tissue	Mavlyutov et al., 2016
Nucleus Nucleoplasmic reticulum Plasma membrane subsurface ER cisternae	Immuno-electron microscopy	NSC34 cells (produced by fusion of motor neuron enriched, embryonic mouse spinal cord cells with mouse neuroblastoma), Sigmar1-null NSC34 cells	Endogenous Sigmar1 stained with antibody as reported in Ramachandran et al. (2007), Exogenously expressed full length Sigmar1-GFP-APEX2 (ascorbate peroxidase 2) fusion construct were enhanced using 3, 3′-diaminobenzidine (DAB) incubation	Reported at (Ramachandran et al., 2007)	Mavlyutov et al., 2017
i. ER, Mitochondria associated ER membranes (MAM) ii. Cholesterol-containing giant unilamellar vesicles (GUVs)	Immuno-fluorescence microscopy	HEK293T cells	i. Exogenously expressed GFP-tagged human Sigmar1 colocalization assessed with mCherry tagged Sec16β protein and immunofluorescence staining of mitochondrial outer membrane protein Tom20 ii. GFP-tagged Sigmar1 fusion constructs	i. and ii. Exogenous overexpression of GFP-tagged fusion constructs	Zhemkov et al., 2021

## Physiological and Pathological Role of Sigmar1

An enormous amount of studies in the current literature have attempted to elucidate Sigmar1’s molecular role under physiological and pathological conditions in different organs. Sigmar1 global knockout (Sigmar1^–/–^) mouse models were used to explore Sigmar1’s physiological functions’ in different organs. To date, two separate lines of Sigmar1^–/–^ mice reported were generated by gene targeting (Oprs1tm1Lmon/Oprs1tm1Lmon) (Langa et al., 2003) and gene trapping [Oprs1Gt(IRESBetageo)33Lex/Oprs1Gt(IRESBetageo)33Lex]^2^. Both of these Sigmar1 homozygous knockout mouse lines were viable and fertile. They did not display any overt phenotype compared with their wild-type littermates. However, all these studies were limited by cursory observations of limited sample sizes from mouse strains with mixed genetic backgrounds (Langa et al., 2003). Extensive studies of this Sigmar1^–/–^ mouse reported the development of multiple pathological phenotypes: locomotor defects (Langa et al., 2003), significant nerve denervation (Bernard-Marissal et al., 2015), loss of motor neurons (Bernard-Marissal et al., 2015), and age−dependent motor phenotype (Watanabe et al., 2016). Studies also demonstrated the development of a depressive-like phenotype (Sabino et al., 2009a) and a gender-related anxiety, depressive-like and memory related alterations in the Sigmar1^–/–^ mouse (Chevallier et al., 2011). Extensive research using Sigmar1 ligands (agonists and antagonists) revealed Sigmar1’s roles in several pathological conditions in different organs summarized below:

### Sigmar1 in Cardiovascular Pathophysiology

The presence of Sigmar1 in the heart was initially reported using ligand binding assays (Ela et al., 1994; Novakova et al., 1995), and since then, pharmacologic targeting has led to hypotheses concerning the potential importance of Sigmar1 protein in the heart (Tagashira et al., 2010, 2011; Bhuiyan and Fukunaga, 2011). All studies to date have been limited to pharmacologic approaches using less selective ligands for Sigmar1 due to the unavailability of genetic models to study the functionality of Sigmar1 in the heart (Tagashira et al., 2010, 2011; Bhuiyan and Fukunaga, 2011). Subsequent studies from our group using Western blot analysis of Sigmar1 protein levels in whole-cell extracts from the thoracic aorta, left ventricle, and right ventricle of rats has revealed ubiquitous expression of Sigmar1 in the major components of the cardiovascular system (Bhuiyan and Fukunaga, 2009, 2011). We recently reported the development of cardiac contractile dysfunction and cardiac fibrosis in Sigmar1 null mice with aging (Abdullah et al., 2018). Hearts of Sigmar1^–/–^ mice developed significant accumulations of irregularly shaped mitochondria and defects in mitochondrial respiratory function. We demonstrated a potential molecular function of Sigmar1 in regulating the mitochondrial bioenergetics that are essential to maintain normal cardiac contractile function (Abdullah et al., 2018). Extensive research has explored the effects of Sigmar1 ligands (agonists and antagonists) on the cardiovascular system using different *in vitro* and *in vivo* cardiac injury models as summarized below:

#### Cardiac Contractility

Initial studies done to characterize Sigmar1 showed that cardiomyocytes exhibit sigma receptor ligand-binding sites, and that several of these Sigmar1 ligands may alter cardiac contractility. Among these Sigmar1 ligands, (+)−3-PPP, (+)-pentazocine, and haloperidol altered the contractility, calcium influx, and rhythmic activity of cultured cardiomyocytes (Ela et al., 1994, 1996; Novakova et al., 1995; Monassier et al., 2007). Several of these Sigmar1 ligands showed ionotropic action on isolated neonatal and adult cardiomyocytes (Novakova et al., 1995). A direct interaction has also demonstrated between the Sigmar1 and human Ether-à-go-go-Related Gene (hERG) that promotes hERG protein level in n myeloid leukemia and colorectal cancer cells (Crottes et al., 2016). Sigmar1 increased hERG current density via a regulation of channel subunit maturation and stability in a chronic myeloid cell line (K562), HEK-293 cells, and Xenopus oocytes (Crottes et al., 2011). The hERG channel is a voltage-dependent K+ channel that regulates cardiac repolarization (Trudeau et al., 1995), but the role of Sigmar1 or Sigmar1 ligands in hERG channel activity in cardiomyocytes has never been studied. It has also been shown that both Sigmar1 agonists (SKF-10047 and (+)-pentazocine) and antagonists (haloperidol and ditolylguanidine) reversibly inhibited Na(v)1.5 channels to varying degrees in HEK-293 cells and COS-7 cells (Johannessen et al., 2009). However, all these four Sigmar1 ligands four ligands also inhibited Na(+) current in neonatal mouse cardiac myocytes (Johannessen et al., 2009). Sigmar1 was also involved in the major Ca^2+^ influx pathway through inhibiting store-operated Ca^2+^ entry (SOCE) and reducing the Ca^2+^ content of the intracellular stores in HEK cells and Sigmar1 expressed HEK cells (Srivats et al., 2016). Stable expression of a Sigmar1 in HEK cells and treatment with Sigmar1 agonists [(+) SKF10047] in CHO cells inhibited SOCE (Srivats et al., 2016). In contrast, Sigmar1 siRNA knockdown and treatment with Sigmar1 antagonists in CHO cells enhanced SOCE (Srivats et al., 2016). Studies demonstrated that haloperidol treatment is frequently accompanied by cardiovascular side effects, including QT interval prolongation and the occurrence of even lethal arrhythmias. Haloperidol treatment in guinea pigs significantly decreased the relative heart rate and prolonged QT interval of the isolated hearts from the haloperidol-treated animals. These effects were associated with the increased expression of Sigmar1 and ITPR (type 1 and type 2) in the atria of haloperidol-treated animals (Stracina et al., 2015). However, Sigmar1 ligands (DTG, PB28, and (+) SKF10047) inhibited Na+ (Nav) channels activity in Sigmar1 siRNA knockout HEK-293 cells indicating Sigmar1 independent effect in the Na+ activity of these ligands (Johannessen et al., 2009, 2011). Similarly, Sigmar1-independent inhibition of the Kv2.1 channel was achieved by sigma ligands (both agonists and antagonists) using Kv2.1-overexpressing HEK-293 cells with and without CRISPR/Cas9 Sigmar1 knockout (Liu et al., 2017). Therefore, Sigmar1 ligands may affect various ion channels via Sigmar1 as well as through a direct action of the ligand on the ion channel function. Overall, ion channel modulation by different Sigmar1 ligands affecting *in vitro* cell contractility was inconsistent. The molecular mechanisms of Sigmar1 interactions and direct involvement with these ion channels remained unknown.

#### Cardiac Hypertrophy

Extensive studies have been focused on the effects of Sigmar1 ligands (both agonists and antagonists) in heart tissues to explore the pathophysiological role of Sigmar1. Temporal study performed to demonstrate the time-dependent changes in Sigmar1 protein levels in the heart showed a significant negative linear correlation with the development of cardiac dysfunction in pressure overload-induced (PO) or transverse aortic constriction (TAC)-induced cardiac hypertrophy (Bhuiyan et al., 2010; Tagashira et al., 2010). Moreover, cardiac hypertrophy in mice induced by aortic banding also exhibit reduced expression levels of Sigmar1 protein in the brain and depression-like behavior, along with the development of impaired cardiac function (Ito et al., 2011). Sigmar1 activation using agonists has been shown to elicit cardioprotection in these rodent models of cardiac hypertrophy and heart failure (Bhuiyan and Fukunaga, 2009, 2011; Bhuiyan et al., 2010, 2011a,b, 2013; [citeskum]BR294,BR293,BR295,BR297,BR291,BR292[citeekum][Bibr B302][Bibr B298]). A number of studies have shown that stimulation of Sigmar1 using its agonists (e.g., dehydroepiandrosterone and fluvoxamine) elicit protective effects against PO-induced cardiac hypertrophy in ovariectomized rats and TAC-induced cardiac hypertrophy in mice (Bhuiyan and Fukunaga, 2009; Bhuiyan et al., 2010; Tagashira et al., 2010). These studies demonstrated that Sigmar1 activation by the agonist ameliorates cardiac hypertrophy and contractile dysfunction by activating the Akt-eNOS signaling pathway (Bhuiyan et al., 2010; Tagashira et al., 2010). This protective effect of Sigmar1 activation (by the use of its agonists) was ablated using Sigmar1 antagonist (NE-100 and haloperidol) (Bhuiyan and Fukunaga, 2009; Bhuiyan et al., 2010; Tagashira et al., 2010). In association with the activation of Akt-eNOS signaling, Sigmar1 activation by agonist also restored TAC-induced alterations in mitochondrial calcium mobilization and ATP production (Tagashira et al., 2013a, c, 2014a). Studies also showed Sigmar1 agonists could restore TAC-mediated disrupted interaction of Sigmar1 with ITPR and negatively regulate ryanodine receptors (Tagashira et al., 2013a, 2014a). However, Sigma1 inhibition by treatment with antagonists aggravated cardiac pathology with aggravation of impaired mitochondrial calcium mobilization, decreased ATP production, increased autophagosome accumulation, and mitochondrial dysfunction with increased mitochondrial fragmentation (Shinoda et al., 2016).

Several studies have demonstrated that the neurosteroid dehydroepiandrosterone (DHEA) serves as an endogenous ligand for Sigmar1, and DHEA treatment ameliorated PO-induced cardiac hypertrophy in ovariectomized rats (Bhuiyan and Fukunaga, 2009; Bhuiyan et al., 2011a; Tagashira et al., 2011). Upregulation of the Sigmar1 protein levels following fluvoxamine and DHEA treatments has been suggested to be responsible for Sigmar1’s cardioprotective action. Several studies using different Sigmar1 ligands showed that these ligands have different effects on Sigmar1 expression. In rats, chronic treatment with the Sigmar1 ligand E-5842 increased Sigmar1 mRNA expression in the brain (Zamanillo et al., 2000), whereas chronic treatment with imipramine decreased levels of Sigmar1 binding sites in the brain (Shirayama et al., 1993). Similarly, chronic haloperidol (a Sigmar1 antagonist) treatment promoted a reduction of Sigmar1 binding sites (Inoue et al., 2000). On the other hand, treatment with the Sigmar1 antagonist NE-100 did not alter Sigmar1 expression in the heart *in vivo* (Tagashira et al., 2010). Moreover, a combination of NE-100 with fluvoxamine nullified fluvoxamine-mediated anti-hypertrophic effects without altering the protein levels of Sigmar1 in the heart (Tagashira et al., 2010). Apparently, these differences in the modulation Sigmar1 mRNA expression and protein level by ligands result from the different methodologies used to examine Sigmar1, including *in vivo* vs. *in vitro* tests and binding assays vs. immunodetection. However, the direct role of Sigmar1 using genetic models has never tested in these cardiac-injury models.

#### Myocardial Infarction

It has also been suggested that Sigmar1 ligands play a potential cardioprotective role in ischemia/reperfusion (I/R) injury. Treatment with Sigmar1 ligand afobazole prevented the development of pathologic remodeling of the myocardium, maintained its inotropic function, and decreased the plasma level of brain natriuretic peptide in a rat model of myocardial infarction. Interestingly, afobazole treatment down-regulated the mRNA expression of angiotensin, vasopressin, glucocorticoid receptor, and Epac2 protein level in the infarcted myocardium (Kryzhanovskii et al., 2018). Another study also showed delayed cardioprotective effects of afobazole, evaluated by using echocardiography in an experim ental myocardial infarction model (rat model of acute myocardial ischemia) (Kryzhanovskii et al., 2017). It has been proposed that the cardiotropic effects of the anxiolytic afobazole were associated with Sigmar1 agonistic effects in cardiomyocytes (Kryzhanovskii et al., 2017, 2018). However, a recent study showed that chronic Sigmar1 activation ameliorated ventricular remodeling and decreased susceptibility to ventricular arrhythmias after myocardial infarction in rats (Fo et al., 2020). Sigmar1 activation following treatment with fluvoxamine improved cardiac function through reduced susceptibility to ventricular arrhythmias, mitigated myocardial fibrosis, lightened sympathetic remodeling and electrical remodeling, and upregulated Sigmar1 protein levels (Fo et al., 2020). Fluvoxamine also significantly prolonged the ventricular effective refractory period, shortened action potential duration, and reduced susceptibility to ventricular arrhythmias after MI (Fo et al., 2020). Similarly, treatment with a Sigmar1 agonist (PRE-084) in rats with I/R injuries improved cardiac hemodynamic parameters, including LV pressure development and left ventricular systolic pressure (Gao et al., 2018). Mechanistically, the protective effect of PRE-084 was associated with the reduction of apoptotic cell death with increased Bcl-2 levels and decreased Bax levels in cardiomyocytes. Sigmar1 dependent activation of the PI3K/Akt/eNOS signaling pathways has been suggested to inhibit I/R injury-induced apoptotic cell death (Gao et al., 2018). A recent study in MI mice showed that a decreased brain Sigmar1 played a vital role in the coexistence of increased HF via sympathoexcitation and mental disorders, such as depression or cognitive impairment (Ito et al., 2013). Interestingly, intracerebroventricular infusion of PRE084 in MI mice improved both mental disorder and cardiac function with lowered sympathetic activity. These protective effects were associated with the PRE084 induced recovery of the Sigmar1 expression in both the hypothalamus and hippocampus (Ito et al., 2013).

#### Atrial Fibrillation

Stimulation of Sigmar1 has also been shown to exhibit cardioprotection in tachycardia, atrial fibrillation, and asphyxia cardiac arrest. Sigmar1 agonist-mediated activation of Sigmar1 decreased the duration of stress-induced tachycardia without altering the peak heart rate in rats (Delaunois et al., 2013). This protective effect of Sigmar1 agonists was abrogated by Sigmar1 antagonists (Delaunois et al., 2013). Similar to the effects of Sigmar1 in ventricles, inhibition of Sigmar1 by treatment with antagonists altered atrial electrophysiology, reducing effective refractory period, action potential duration, and leading to increased inducibility and time of atrial fibrillation (Ye et al., 2019). Furthermore, inhibition of Sigmar1 by antagonists resulted in increased atrial fibrosis and reduced the levels of connexin 40 (a gap junction protein) (Ye et al., 2019), leading to slow conduction of electrical impulses across atria (Ye et al., 2019). Treatment with a Sigmar1 agonist reversed these effects rescuing the effects of Sigmar1 inhibition (Ye et al., 2019). Similarly, Sigmar1 activation by selective Sigmar1 ligands also protected the depression-induced atrial fibrillation (Liu et al., 2018b, 2019).

#### Vascular Disease

Studies have also suggested that the presence of Sigmar1 in aortic vasculature and its involvement in vascular remodeling was induced by pressure overload. Decreased expression of Sigmar1 protein levels was observed in aortic cell lysate after the PO model of cardiac injury in rats and the TAC model of cardiac injury in mice. The decreased Sigmar1 protein levels were associated with inhibition of the Akt-eNOS signaling pathway in the aorta. Activation of Sigmar1 in these models of aortic injury activated the Akt-eNOS mediated signaling, rescued the aortic injury, and resulted in aortic relaxation (Tagashira et al., 2013b). In fact, Sigmar1 activation by DHEA and fluvoxamine restored Akt activity, ameliorated impaired eNOS expression, and eNOS phosphorylation in the thoracic aorta after cardiac injury (Bhuiyan et al., 2010, 2011a,b; Tagashira et al., 2010, 2011).

Recently, the role of Sigma receptor in angiogenesis was demonstrated by using (±)-haloperidol metabolite II valproate ester [(±)-MRJF22], which was a prodrug of haloperidol metabolite II (Sigmar1 antagonist/Sigmar2 agonist ligand) obtained by conjugation to valproic acid (histone deacetylase inhibitor) via an ester bond (Olivieri et al., 2016). (±)-Haloperidol metabolite II valproate ester [(±)-MRJF22] exhibited an antiangiogenic effect, significantly reduced cell viability, endothelial cell migration, and tube formation in vascular endothelial growth factor A (VEGF-A) stimulated human retinal endothelial cell cultures (Olivieri et al., 2016). However, the direct role of Sigmar1 in endothelial cell proliferation, migration, angiogenesis, and function remained elusive. Further studies are required to demonstrate the clinical efficacy of Sigmar1 ligands (agonists or antagonists) in endothelial cell pathologies in humans.

The lymphatic system is fundamentally important to several pathologies, including cardiovascular disease, edema, infection, Crohn’s disease, cancer, and obesity (Mortimer and Rockson, 2014). Sigmar1 mRNA and protein has been detected in lysates from isolated rat mesenteric collecting lymphatics, and Sigmar1 localization has been observed in the lymphatic endothelium using immunofluorescence confocal microscopy (Trujillo et al., 2017). Sigmar1 activation by the anxiolytic afobazole (an agonist of the Sigmar1) reduced lymphatic pump function elicited by an elevation in normalized end-systolic diameter, resulting in the decreased normalized amplitude of contraction, ejection fraction, and fractional pump flow (FPF) in isolated rat mesenteric lymphatics (Trujillo et al., 2017). Although simultaneous treatment with several Sigmar1 antagonists (BD 1047, BD 1063, and SM-21) reduced the effects of afobazole on lymphatic contraction, suggesting the involvement of Sigmar1, afobazole has been reported to be a mixed Sigmar1/Sigmar2 agonist (Katnik et al., 2016) that also has a high affinity for the melatonin MT1 receptor (Seredenin and Voronin, 2009). Afobazole-induced changes in lymphatic pump function were mediated via endothelial NO production in cultured lymphatic endothelial cells (Trujillo et al., 2017). However, it has also been suggested that Afobazole-induced NO-independent effects, as afobazole treatment in the presence of NOS inhibitor L-NAME led to a decrease in the normalized end-diastolic diameter of the isolated lymphatic vessel (Trujillo et al., 2017). Recently, Sigmar1’s role in lymphatic endothelial barrier function has been demonstrated by a study showing the contribution of Sigmar1 to basal lymphatic endothelial barrier function, potentially through the enhancement of glycolytic energy production in cultured adult human dermal lymphatic endothelial cells (Motawe et al., 2020). Despite all these pharmacologic data collected using non-selective ligands, the molecular role of Sigmar1 in the pathophysiology associated with the lymphatic system remains unknown. Therefore, future studies are required to determine the role of Sigmar1 in the lymphatic system, which could potentially be useful for individuals with lymphatic system disorders.

#### Drug-Induced Cardiomyopathy

We recently reported a potential protective role for Sigmar1 in methamphetamine-induced cardiomyopathy, where methamphetamine reduced Sigmar1 protein levels in mice, rats, and humans (Abdullah et al., 2020). Methamphetamine use in humans, rats (self-administered), mice (Binge-and-Crash model of injection) resulted in increased collagen and fibrosis, cardiac hypertrophy, mitochondrial dysfunction with altered morphology, dynamics, and reduced bioenergetics. Moreover, methamphetamine consumption reduced the levels of Sigmar1 correlated with methamphetamine-induced cardiac and mitochondrial dysfunction (Abdullah et al., 2018, 2020).

#### Maladaptive ER Stress

Recently, we also reported a cardioprotective role for Sigmar1 against maladaptive ER stress (Alam et al., 2017). An array of pathological stress responses that lead to cardiovascular disease results in ER stress characterized by the accumulation of unfolded and misfolded proteins. C/EBP-homologous protein (CHOP) is a ubiquitously expressed stress-inducible transcription factor whose expression is robustly induced by maladaptive endoplasmic reticulum (ER) stresses in a wide variety of cells. Sigmar1-siRNA knockdown in neonatal rat ventricular cardiomyocytes (NRCs) has been found to significantly increase the expression of CHOP and induced cellular toxicity by sustained activation of ER stress in cardiomyocytes. Conversely, adenovirus-mediated Sigmar1 overexpression decreased the expression of CHOP and significantly decreased cellular toxicity in cardiomyocytes. Sigmar1 overexpression significantly increased inositol requiring kinase 1α (IRE1α) phosphorylation and increased spliced X-box-binding proteins (XBP1s) expression as well as nuclear localization. In contrast, Sigmar1 knockdown significantly decreased IRE1α phosphorylation and decreased XBP1s expression as well as nuclear transport. Overall, Sigmar1-dependent activation of IRE1α-XBP1s ER-stress response pathways was associated with inhibition of CHOP expression and suppression of cellular toxicity. Therefore, Sigmar1 functions as an essential component of the adaptive ER-stress response pathways eliciting cellular protection in cardiomyocytes (Alam et al., 2017).

Despite the existence of knockout mice, all studies to date have been limited to pharmacologic approaches using less selective ligands for Sigmar1 (Bhuiyan et al., 2010; Bhuiyan and Fukunaga, 2011; Tagashira et al., 2011). The role of Sigmar1 in the heart has remained elusive, as all previously described Sigmar1 ligands [such as fluvoxamine (Omori et al., 2010), sertraline (Kim et al., 2016), (+) pentazocine (Hernandez and Appel, 1979), haloperidol (Chertkow et al., 2007), and cutamesine (SA4503) (Matsuno et al., 1996)] involve serotonin reuptake inhibitors (SSRIs) and also have a wide affinity for other receptors (Hayashi et al., 2011; Niitsu et al., 2012). Although approximately 35 publications have dealt with Sigmar1’s possible functions in cardiomyocytes, all current studies have been correlative, limited to pharmacologic approaches using less selective ligands (e.g., SSRIs), and the molecular mechanisms has not been unexplored (Fontanilla et al., 2009; Johannessen et al., 2009; Crottes et al., 2011; Amer et al., 2013). Sigmar1 has a significant therapeutic potential to treat the cardiovascular disease as reflected by two Sigmar1 ligands already in clinical trials: cutamesine (SA4503) for ischemic stroke (Phase II) (Urfer et al., 2014) and sertraline for depression in patients with heart failure (SADHART-CHF) (Serebruany et al., 2003, 2005; Swenson et al., 2003; Glassman et al., 2006; Jiang et al., 2008, 2011; O’Connor et al., 2010; Xiong et al., 2012, 2015). However, a direct role for cardiac Sigmar1 has not been defined. A major barrier to understanding the molecular functions of Sigmar1 is the lack of organ-specific genetic mouse models (either Tg or knockout) and selective ligands. Therefore, achieving an understanding of the molecular function of Sigmar1 would allow us to design selective Sigmar1 activators, which could be used to therapeutically to prevent cardiomyocytes loss and mitigate the clinical progression of heart failure in patients.

### Neuromuscular Dysfunction

**FIGURE 3:**
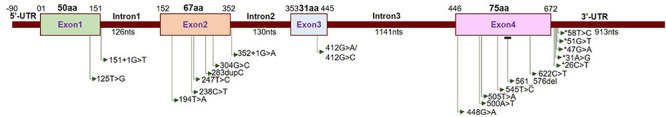
Localization of neuropathy-related mutations in the SIGMAR1 gene. Schematic diagram representing the genetic structure of the SIGMAR1 gene showing the locations of all the mutations related to skeletal muscle pathology.

Neuromuscular disorder comprises a range of conditions that impair the functioning of the muscles, either directly due to pathologies of the voluntary muscle or indirectly due to pathologies of the peripheral nervous system or neuromuscular junctions. Progressive muscle weakness is the predominant condition associated with these disorders. Extensive studies have identified several recessive mutations in SIGMAR1 in association with a range of neuromuscular disorders, including amyotrophic lateral sclerosis (ALS) (Tagashira et al., 2014b; Fukunaga et al., 2015), ALS with or without frontotemporal lobar degeneration (Luty et al., 2010; Ullah et al., 2015), juvenile ALS (Al-Saif et al., 2011; Watanabe et al., 2016), distal hereditary motor neuropathy (dHMN) (Li et al., 2015; Gregianin et al., 2016; Horga et al., 2016; Lee et al., 2016; Almendra et al., 2018; Nandhagopal et al., 2018), frontotemporal lobe degeneration (FTLD) (Li et al., 2015; Gregianin et al., 2016; Horga et al., 2016; Lee et al., 2016; Almendra et al., 2018; Nandhagopal et al., 2018), and silver-like syndrome (Horga et al., 2016) as summarized in Table 2 and Figure 3.

**TABLE 2:** Genetic and clinical features of patients with SIGMAR1 mutations.

**Genotype**	**Protein**	**Ethnic origin**	**Diagnosis**	**Muscle weakness onset age**	**Muscle weakness**	**Knee Jerks**	**Babinski Response**	**Brain MRI**	**Citations**
c.505T > A/.622C > T	p.T169R/p.R208W	Japanese	ALS	80y	+	Brisk	Present	Normal	Izumi et al., 2018
c.283dupC	p.L95Pfs*29	Hispanic	Juvenile ALS	5y	+	Brisk	Present	Normal	Watanabe et al., 2016
c.304G > *C*	p.E102Q	Saudi	Juvenile ALS	1–2y	+	Brisk	N/A	Normal	Al-Saif et al., 2011
*c.58T > C	3′-UTR	Korean	Sporadic ALS	55y	+	N/A	N/A	N/A	Kim et al., 2014
*31A > G	3’-UTR	Pakistani	Juvenile ALS	30–36yrs	+	Brisk	N/A	Normal	Ullah et al., 2015
c.412G > A	p.E138K	Italian	HMN	10—18y	+	Brisk	N/A	Mild cerebral atrophy	Previtali et al., 2019
c.247T > C/c.545T > C	p.F83L/p.L182P	German/French	HMN	34y	+	Brisk	Present	Normal	Ma et al., 2020
c.412G > *C*	p.E138Q	Italian	HMN	9–12y	+	Brisk	Present	Normal	Gregianin et al., 2016)
c.448G > *A*	p.E150K	Italian	HMN	Infancy	+	Brisk	Present	Normal	Gregianin et al., 2016
c.238C > *T*	p.Q80*	Omani	dHMN	1–11y	+	Brisk	Present	Normal	Nandhagopal et al., 2018
c.500A > *T*	p.N167I	Jordanian	dHMN	6–10y	+	Brisk	Present	Normal	Christodoulou et al., 2000
c.561_576del	p.D188Pfs*69	Portuguese	dHMN	4y	+	Absent	N/A	Normal	Almendra et al., 2018
*51G > T	3′-UTR	Australian	FTLD	No info	No info	No info	No info	No info	Luty et al., 2010; Belzil et al., 2013
*26C > T	3′-UTR	Australian	FTLD	No info	No info	No info	No info	No info	Luty et al., 2010; Belzil et al., 2013
*47G > A	3′-UTR	Polish	FTLD	No info	No info	No info	No info	No info	Luty et al., 2010; Belzil et al., 2013
c.194T > *A*	p.L65Q	French/British	Silver-like syndrome	3y	+	Brisk	Present	Normal	Horga et al., 2016

“*” means 3’-UTR.

Most of the genetic studies of Sigmar1 havedemonstrated an association between Sigmar1 mutations and ALS pathology. The clinical hallmarks of ALS pathology include progressive muscle wasting, speech and swallowing difficulties, fasciculation, altered reflexes, spasticity, and death due to respiratory complications (Loeffler et al., 2016). Juvenile cases of ALS have been associated with a missense mutation (c.304G > C, p.E102Q) (Al-Saif et al., 2011) and a frameshift mutation (c.283dupC, p.L95 fs) in Sigmar1 (Watanabe et al., 2016). Progressive development of skeletal muscle pathology was observed in E102Q mutations bearing patients, including weakness of the hand and forearm muscles (at the age of 9 to 10 years) leading to paralysis of forearm extensors and triceps. These patients had no respiratory or bulbar muscle weakness and demonstrated normal sphincteric, sensory, and cerebellar functions (Al-Saif et al., 2011). Similarly, the patient with the L95 fs mutations developed progressive muscle weakness with significant atrophy of distal muscles with development of pes cavus and wasting of the calf muscles and the intrinsic muscles of the hands (Watanabe et al., 2016). Interestingly, examination of a biopsy of vastus lateralis muscle showed severe type II fiber predominance with scattered angular esterase positive fibers, and also showed intense staining with nicotinamide adenine dinucleotide tetrazolium reductase (NADH−TR) (Watanabe et al., 2016). Patients bearing these mutations showed normal brain and spinal cord magnetic resonance imaging (MRI) (Al-Saif et al., 2011; Watanabe et al., 2016).

These clinical skeletal muscle phenotypes, all of which were observed in Sigmar1 mutation-bearing patients, have also been observed in patients with distal hereditary motor neuropathy (dHMN). In fact, several of the truncations/deletions or point mutations in Sigmar1 have also beenreported in association with the development of dHMN (Li et al., 2015; Gregianin et al., 2016; Horga et al., 2016; Lee et al., 2016; Almendra et al., 2018; Nandhagopal et al., 2018). The dHMN comprise a heterogeneous group of diseases having the common feature of slowly progressive, symmetrical, and distal-predominant neurogenic weakness and amyotrophy. All dHMN patients with the Sigmar1 mutations manifest identical clinical features: progressive muscle wasting/weakness in the lower and upper limbs without sensory loss (Li et al., 2015; Gregianin et al., 2016; Horga et al., 2016; Lee et al., 2016; Almendra et al., 2018; Nandhagopal et al., 2018) accompanied by normal brain and spine MRI (Gregianin et al., 2016).

Studies have also shown an association of Sigmar1 mutations in the 3′−untranslated region with the frontotemporal lobar degeneration (FTLD)-motor neuron disease (MND). Sigmar1 normally localizes to cytoplasmic membranes in healthy individuals, while in the c.672^∗^51G > T carriers showed intense Sigmar1 immunoreactivity in the nucleus dentate granule and CA1 pyramidal cells. However, the details of the clinical features in these patients remain unknown. Patients bearing a homozygous missense variant (c.194T > A, p.Leu65Gln) of Sigmar1 have been associated with autosomal recessive Silver-like syndrome (Horga et al., 2016). The clinical feature of this Sigmar1 mutation-bearing patient includes bilateral foot drop and frequent falls (at age 3 years), and development of progressive muscle weakness and atrophy in the lower limbs. This patient developed clawed hands with no fixed contractures, bilateral finger and foot drop, knee bobbing, marked muscle atrophy from mid-forearms and knees down, and weakness of wrist extension at the age of 17 years. However, the patient had normal intellect, no sensory symptoms, and no sphincter problems with normal brain and spinal cord MRIs.

Despite the evidence in these reports, proof of a direct association between mutations in Sigmar1 and human diseases remains elusive, as this association has only been identified in small, isolated families, with limited genetic and functional studies. Functional studies to determine molecular mechanism showed that ALS associated Sigmar1 mutations (p.E102Q and p.L95 fs) (Al-Saif et al., 2011; Watanabe et al., 2016) are uniformly unstable and non−functional when expressed in Neuro2a (N2a) cells, suggesting a role of Sigmar1’s loss of function in ALS (Al-Saif et al., 2011; Watanabe et al., 2016). Moreover, expression of the Sigmar1 E102Q carrying mutation in Drosophila (which lacks a Sigmar1 homolog) alters locomotor activity and eye development (Couly et al., 2020b). Whereas fu+nctional studies using two of dHMN associated mutations (p.E138Q and p.E150K) in several neuronal cell lines (two human neuroblastoma cell lines, SH-SY5Y and SK-N-BE, and the murine motor neuron-like NSC-34 line) suggested the pathogenicity of the mutations may involve the alterations in ER-mitochondria tethering, calcium homeostasis, and autophagy. The presence of the c.672^∗^26C > T, c.672^∗^47G > A, and c.672^∗^51G > T mutations within the 3′−UTR of SIGMAR1 affect transcript stability resulting in increased Sigmar1 transcript in human neuroblastoma SK−N−MC and HEK-293 cells (Luty et al., 2010). Though studies using Sigmar1 global knockout mice provided a molecular tool to understand the physiological function of Sigmar1 (Langa et al., 2003), these mice did not show any pathological phenotype associated with the human diseases observed in Sigmar1 mutation bearing patients. The neuronal dysfunction reported in Sigmar1^–/–^ mice were locomotor defects (Mavlyutov et al., 2010), nerve denervation (Bernard-Marissal et al., 2015), loss of motor neurons (Bernard-Marissal et al., 2015), age−dependent motor dysfunction (Watanabe et al., 2016), and development of depressive-like behavior (Langa et al., 2003; Sabino et al., 2009a).

The most common clinical feature observed in patients with Sigmar1 mutation is muscle weakness caused possibly as a result of myofiber injury or by motor neuron injury resulting in denervation. However, the physiological function of Sigmar1 in skeletal muscle has never been studied and remains elusive.

### The Involvement of Sigmar1 in Physiological and Pathological Conditions in the Brain

Since the discovery of Sigmar1, most studies have focused on elucidating the role of Sigmar1 under physiological and pathological conditions in the brain. Studies have demonstrated that the absence of Sigmar1 in Sigmar1^–/–^ mice affected a wide range of brain functions (Couly et al., 2020a), including regulation of cognition and memory (Chevallier et al., 2011), motor activity (Bernard-Marissal et al., 2015), psychiatry-related behaviors (Chevallier et al., 2011; Di et al., 2017), sensory system and pain (Cendan et al., 2005). However, studies carried out to demonstrate the role of Sigmar1 in memory regulation using the Sigmar1 null mice resulted in inconsistent data. Behavioral studies using Sigmar1 null mice in different age groups (7–48 weeks) and of both sexes showed an array of effects ranging from no impact to a loss of long-term memory without alterations in short-term memory (reviewed in Couly et al., 2020a). Studies of the alteration in motor-related behaviors included movements from spontaneous locomotion to motor coordination and muscle strength. Sigmar1 null mice did not show any affects to spontaneous locomotion, as demonstrated by the open-field test and Y-maze test (Langa et al., 2003; Chevallier et al., 2011). However, the absence of Sigmar1 impairs motor coordination in an age-dependent manner, as shown by the lower motor coordination scores of older Sigmar1 null mice during the rotarod test. Similarly, Sigmar1 null mice showed reduced muscle strength compared to age-matched controls (Bernard-Marissal et al., 2015). The absence of Sigmar1 also resulted in an abnormal swimming patterns in Sigmar1 null mice without altering the swimming efficacy and speed (Chevallier et al., 2011; Di et al., 2017). Sigmar1 has a prominent effect on the psychiatric behaviors in mice, including depression and anxiety. The absence of Sigmar1 results in an increased depressive phenotype as shown by increased immobility in forced swimming test and tail suspension test (Chevallier et al., 2011; Sha et al., 2015). Additionally, a lack of Sigmar1 showed normal anxiety-like behavior as shown during the elevated plus maze test and light-dark transfer test (Sabino et al., 2009a).

Since the discovery of Sigmar1, alterations in the it’s function have been reported to associate with the development of neurodegenerative diseases, including Alzheimer’s disease (AD), Parkinson’s disease (PD), and Huntington’s disease (HD). Broadly, Sigmar1 activation using ligands elicit potent neuroprotective effects, promotes neuronal survival, and restores neuronal plasticity to slow disease progression, whereas dysfunction in Sigmar1 may worsen the progression of neurodegenerative diseases. Here, we summarize the different neurodegenerative diseases associated with the dysfunction of Sigmar1.

#### Role of Sigmar1 in the Pathogenesis of Alzheimer’s Disease (AD)

Alzheimer’s disease is a progressive neurodegenerative disease characterized by an accumulation of protein aggregates (e.g., Aβ-containing amyloid plaques and tau-derived neurofibrillary tangles), memory loss [both short and long term (late-stage)], cognitive deficits with impaired reading, writing, and learning abilities, behavioral changes (increased aggression, loss of empathy), loss of motor coordination and exhaustion (late-stage) (Förstl and Kurz, 1999). Earlier studies have demonstrated an association between Sigmar1’s polymorphism and the risk of developing AD (Feher et al., 2012). Further genetic studies have shown an association between Sigmar1’s genetic polymorphisms and apolipoprotein E (APOE), which influences the severity of AD across multiple ethnic populations (Huang et al., 2011). Additional studies using postmortem samples from AD patients revealed reduced Sigmar1 binding sites (Jansen et al., 1993). Notably, *in vivo* brain imaging using [11C]-SA4503 showed a reduced Sigmar1 density in the frontal, temporal, occipital lobes, cerebellum, and thalamus of early stage AD patients (Mishina et al., 2008; Toyohara et al., 2009). Consisted with these observations in humans, both knockout of Sigmar1 and pharmacological inactivation with the antagonist (NE-100) aggravated Aβ25–35-induced toxicity with the concurrent development of learning impairment, oxidative stress, and BDNF alteration in animal models of AD (Maurice et al., 2018). Extensive studies have shown that treatment with Sigmar1 agonists improved cognition in various preclinical animal models, including Aβ25–35 peptide-induced neurodegeneration (Maurice et al., 1998; Meunier et al., 2006b; Villard et al., 2009), cholinergic deficits (Matsuno et al., 1993; Senda et al., 1997; Maurice et al., 2001), aging-induced memory loss (Maurice et al., 1996; Phan et al., 2003), hypoxia, and toxin-induced neurodegeneration (Maurice et al., 1994b, 1999), as well as drug-induced glutamatergic, serotonergic, or calcium channel deficits models of neurodegeneration (Matsuno et al., 1994; Maurice et al., 1994a, 1995). The molecular mechanisms by which Sigmar1 agonists induced protective effects were mediated through the modulation of glutamate release, calcium homeostasis, functional modulation of ion channels, NMDA activity, neuroplasticity, reduction of oxidative stress, and modulation of mitochondrial function (Jansen et al., 1993; Maurice et al., 1998; Uchida et al., 2005; Mishina et al., 2008; Feher et al., 2012). Studies also demonstrated that Sigmar1 ligands (e.g., OZP002, donepezil, ANAVEX2-73) prevented toxicity and memory impairment in pharmacologic and genetic mouse models of AD (Maurice et al., 2019; Ryskamp et al., 2019). Although several studies suggest a protective roles of Sigmar1 ligands in AD pathology (Ryskamp et al., 2019), the direct role for Sigmar1 in AD pathobiology has never been studied using genetic mouse models (organ-specific knockout and transgenic mouse for Sigmar1).

#### Role of Sigmar1 in the Pathogenesis of Huntington’s Disease (HD)

Huntington’s disease is a progressive and inherited neurodegenerative disease exhibiting phenotypes such as motor defects, cognitive decline, and psychiatric symptoms. HD is characterized by the accumulation of huntingtin protein aggregates [caused by a mutation in the huntingtin (Htt) gene]. Huntingtin protein regulates multiple cellular functions, including cell division, vesicle recycling and trafficking, autophagy (aids in cargo recognition), cell survival, and several other functions. Mutation in huntingtin protein disrupts all these cellular functions leading to increased apoptosis, cellular degeneration, impaired autophagic clearance, and dysfunctional vesicle transport. Additionally, mutant huntingtin protein interacts with mitochondria causing mitochondrial dysfunction with altered metabolism and increased reactive oxygen species (ROS) (Zuccato et al., 2010; Saudou and Humbert, 2016). Additionally, mutant huntingtin protein is associated with decreased levels of NF-κB-p65 and activated calpastatin levels (leading to increased ROS levels) (Hyrskyluoto et al., 2013).

The evidence for the Sigmar1’s role in HD pathology was provided by the initial *in vitro* studies in which the expression of N-terminal huntingtin fragment proteins with 120 polyQ repeats or the full-length Htt protein with 75 repeats downregulated Sigmar1 level in neuronal PC6.3 cells. Treatment with Sigmar1 agonist (PRE-084) in this model increased cellular survival and prevented the deleterious effects of Htt (Hyrskyluoto et al., 2013). It has also beenreported that accumulation of Sigmar1 was common to neuronal nuclear inclusions in the brains of patients with five HD, dentatorubral-pallidoluysian atrophy, spinocerebellar ataxia types 1–3, and intranuclear inclusion body disease (Miki et al., 2014). In the cellular model of HD, silencing of Sigmar1 significantly increased the number of nuclear inclusions and caused the accumulation of high-molecular-mass GFP-labeled mutant huntingtin (Miki et al., 2015). Mechanistically, Sigmar1 knockdown studies showed Sigmar1 degrades aberrant mutant huntingtin proteins in the nucleus via activation of the ER-related degradation machinery (Miki et al., 2015). Moreover, a recent study showed that pridopidine (a therapeutic drug for HD) has an affinity for Sigmar1 and acts via Sigmar1 at a nanomolar level. A PET scan study of human HD patients also showed complete Sigmar1 occupancy by pridopidine (Grachev et al., 2020; Battista et al., 2021), and the neuroprotective effects of pridopidine were abolished in Sigmar1 knockout mice (Francardo et al., 2019). Activation of Sigmar1 by pridopidine rescued mitochondrial dysfunction induced by oxidative damage in YAC128 transgenic mice, human HD lymphoblasts, and human HD neural stem cells (NSCs) (Naia et al., 2021). Moreover, early pridopidine treatment was effective in delaying onset of HD-related motor symptoms in YAC128 HD mice (Naia et al., 2021).

#### Role of Sigmar1 in the Pathogenesis of Parkinson’s Disease (PD)

Parkinson’s disease is a slowly progressing brain disease characterized by abnormal locomotion (such as shaking, stiffness, and difficulty in walking, balance, and coordination), and the deposition of Lewy bodies, α-synuclein, ubiquitin, and neurofilaments. Patients with PD exhibit a low Sigmar1 ligand (SA4503) binding potential in the putamen of the brain visualized by PET scan compared to healthy controls (Mishina et al., 2005). Chronic treatment with Sigmar1 agonists (PRE-084 and pridopidine) elicits gradual improvement of parkinsonian-like motor deficits in PD model mice developed by intrastriatal 6-hydroxydopamine (6-OHDA) (Francardo et al., 2014, 2019). Similarly, studies have also shown that PRE-084 treatment in animals reduced neuroinflammation, increased density of dopaminergic fibers in the denervated striatal regions, increased the levels of neurotrophic factors [e.g., brain-derived neurotrophic factor (BDNF) and glial-derived neurotrophic factor (GDNF) in the striatum and nigra], and monoamines (dopamine, DA, and serotonin, 5-HT) (Francardo et al., 2014). In contrast to these studies, treatment with Sigmar1 antagonists (NE 100) and Sigmar1 null mice showed reduced 1-methyl-4-phenyl-1,2,3,6-tetrahydropyridine (MPTP)-induced dopaminergic neurons death and parkinsonism by suppressing N-methyl-d-aspartate receptor (NMDAr) function and dopamine transporter (DAT) expression (Hong et al., 2015). Additionally, inhibition of Sigmar1 by treatment with antagonists (NE-100) prevented neurotoxin-induced neurodegeneration through facilitating TRPC1-mediated calcium influx in SH-SY5Y cells (Sun et al., 2020). As these studies have suggested, either pharmacologic activation/inhibition of Sigmar1 could be useful in slowing down the progression of PD. Robust preclinical studies are required using more clinically relevant mouse models of PD (such as transgenic alpha-synuclein overexpressing mice) to move forward the preclinical research to clinical trials.

Overall, Sigmar1 activation has shown protective effects in different neurodegenerative diseases (AD, HD, PD) through the involvement of different cellular pathway modulation, including mitochondrial function regulation, autophagy, calcium homeostasis regulation, and chaperone function. This makes Sigmar1 a possible target in treating pathologies where modulating Sigmar1 activity could be used as a therapeutic approach in the treatment of neurodegenerative diseases.

### Ischemic Brain Injury

A major therapeutic goal during the subacute and chronic phases of stroke is the enhancement of functional recovery, as a significant number of patients suffer from persistent neurological deficits. Sigmar1 has also been implicated in brain injuries; the expression of Sigmar1 increased in the penumbral or peri-infarct region, making it useful as a molecular marker and therapeutic target in the treatment of acute ischemic stroke (Zhang et al., 2017c). Subsequent studies have shown that pharmacologic activation of Sigmar1 in endothelial cells reduces infarct size, protects blood-brain-barrier integrity, and protective against dementia and learning disabilities (Liu et al., 2018a). Studies have also shown that treatment with Sigmar1 agonists (such PRE-084) reduced infarct volume, neurological deficits, levels of pro-inflammatory cytokines, and enhanced the actions of anti-inflammatory cytokines after embolic stroke in rats (Allahtavakoli and Jarrott, 2011). Similarly, treatment with the Sigmar1 ligand 4-phenyl-1-(4-phenylbutyl)-piperidine (PPBP) decreased cortical infarction volume without altering neurobehavior after transient focal ischemia and prolonged reperfusion in the rat (Harukuni et al., 2000). A preclinical study using permanent middle cerebral artery occlusion in rats showed that chronic treatment with the Sigmar1 agonist cutamesine (SA4503) for a period of 28 days enhanced functional recovery after experimental stroke without affecting infarct size when treatment was initiated within 48 h (Ruscher et al., 2011). A Phase 2 clinical trial was conducted to explore the safety, tolerability, dose range, and functional effects of cutamesine in patients with ischemic stroke (Urfer et al., 2014). *Post hoc* analysis of moderately and severely affected patients (baseline National Institutes of Health Stroke Scale, ≥ 7 and ≥ 10) showed greater National Institutes of Health Stroke Scale improvements compared with placebo (Urfer et al., 2014). Although cutamesine was safe and well-tolerated at the tested dosage levels, the study did not show any significant effects on functional end points in the population as a whole (Urfer et al., 2014).

### Drug Addiction

Extensive research has documented Sigmar1’s involvement in drug addiction pathobiology. In fact, selective Sigma1 ligands modulate monoaminergic systems, particularly dopaminergic as well as serotoninergic systems. Sigmar1 has strong affinities and interacts with several addictive drugs, such as (±)-3,4-methylenedioxymeth-amphetamine (MDMA; derivative of amphetamine), methamphetamine (METH), and cocaine, mediating their locomotor stimulatory and neurotoxic effects (Nguyen et al., 2005; Brammer et al., 2006).

#### Methamphetamine

Sigmar1 has been extensively studies as a possible therapeutic target for use in disrupting the methamphetamine-induced addictive process and toxicity (Sambo et al., 2017, 2018), as studies have reported that methamphetamine binds to Sigmar1 at physiologically relevant concentrations (Ki 2.16 ± 0.25 μM) (Nguyen et al., 2005). Although the molecular consequences of methamphetamine binding to Sigmar1 remain unknown, studies suggest that methamphetamine may exhibit antagonist activity (Hayashi and Su, 2007) and/or act as an inverse agonist for Sigmar1 (Yasui and Su, 2016). Pharmacologic activation of Sigmar1 by treatment with agonists have been shown to attenuate methamphetamine-induced behavioral responses, hyperthermia, and neurotoxicity. Pretreatment with the Sigmar1 agonist PRE-084 decreases methamphetamine-induced psychomotor responses, drug-seeking behavior, and enhancement of the brain reward function (Sambo et al., 2017, 2018). In contrast, studies have also shown that Sigmar1 antagonist, N-[2-(3,4-dichlorophenyl)ethyl]-N-methyl-2-2(dimethylamino)ethylamine (BD1047), exert a protective effect against MDMA-, and methamphetamine-induced locomotion stimulatory effects (Nguyen et al., 2005; Brammer et al., 2006). Despite the evidence from all these studies demonstrating Sigmar1’s role in methamphetamine-induced cellular dysfunction, it remains unknown whether and how Sigmar1 contributes to cellular protection.

#### Cocaine

Sigmar1 had been implicated in the cocaine-induced addictive process and toxicity, and studies have shown that cocaine binds to Sigmar1 (at Asp188) at physiologically reward-relevant concentrations (2–7 μM) (Sharkey et al., 1988; Kahoun and Ruoho, 1992; Matsumoto et al., 2002; Chen et al., 2007). Several studies reported that treatment with selective Sigmar1 antagonists mitigates cellular and behavioral toxicities induced by cocaine, including convulsion and death (Matsumoto et al., 2004, 2014; Robson et al., 2012). Mechanistically, the addictive and neurotoxic actions of cocaine were mediated through Sigmar1 activation, enhancing IP3-dependent Ca2+ signaling (Barr et al., 2015). Cocaine also transcriptionally suppresses the expression of monoamine oxidase B (MAOB) through Sigmar1-dependent recruitment of HDACs (Tsai et al., 2015). The Sigmar1 dependent addictive effect of cocaine was confirmed in studies using Sigmar1 null mice where the absence of Sigmar1 abrogated the suppression of MAOB expression (Tsai et al., 2015). Extensive studies have demonstrated that pharmacologic Sigmar1 antagonists (BD1063, BD1047, NE-100) elicit protection against cocaine-mediated addictive effects on locomotion and neurotoxicity (Maurice et al., 2002; Romieu et al., 2002). However, treatment with the Sigmar1 antagonist, BD1047, did not block cocaine self-administration; however, it did attenuate the cocaine reinstatement.

#### Alcohol

Extensive studies on alcohol use disorder have suggested Sigmar1 as a potential mediator of alcohol reward and reinforcement (Quadir et al., 2019). Several studies have shown that inhibition of Sigmar1 by treatment with Sigmar1 antagonists (BD1063, NE-100) reduced ethanol consumption and ethanol-induced rewarding effects such locomotion stimulation and taste aversion. These effects were reverted by treatment with Sigmar1 agonists in rats (voluntary consumption) and mice (intraperitoneal injection) (Maurice et al., 2003; Sabino et al., 2009b; Blasio et al., 2015). However, Sigmar1 null mice subjected to voluntary ethanol consumption showed increased alcohol drinking with increased taste aversion and hypothermia with no effects on locomotion (Valenza et al., 2016). The contrasting effects of Sigmar1 on ethanol consumption and ethanol-induced locomotor effects observed by different groups might be due to differences in animal models used in the study (mice vs. rats), and the protocol used for ethanol consumption [voluntary vs. intraperitoneal injection (used in Sigmar1 null mice)]. Sigmar1 is also involved in both the chronic effects and withdrawal effects of alcohol consumption, where modulation of Sigmar1 using a selective agonist reduced hyper-responsiveness and mitigate the effects of chronic alcohol consumption induced cognitive decline (Meunier et al., 2006a). Despite these contradictory findings concerning the role of Sigmar1 ligands in alcohol consumption and rewarding effects, all these studies suggested Sigmar1’s involvement in alcohol consumption (acute and chronic) mediated toxic effects. Further studies are required to demonstrate whether modulation of Sigmar1 using selective ligands can be used therapeutically to reverse alcohol consumption-mediated adverse effects.

### Cancer

The role of Sigmar1 has been widely studied in different types of cancers, including prostate cancer, colorectal cancer, breast cancer, and hepatocarcinoma. Clinical studies have shown high levels of Sigmar1 in tumor tissues from breast cancer patients and has been proposed to be used as a clinical marker of breast cancer (Simony-Lafontaine et al., 2000). Similar results were seen in lung cancer patients’ samples where Sigmar1 was secreted by tumor cells and increased the viability of squamous lung cancer cells, and correlated with increased survival of the cancer cells (Mir et al., 2012). Elevated levels of Sigmar1 (mostly cytoplasmic) were also reported in hilar cholangiocarcinoma tissue samples, and correlated with poor prognosis of the patients and their decreased longevity (Xu et al., 2014). Patients with colorectal cancer also exhibit upregulated levels of Sigmar1 depending on the stage of the disease, especially in the upper colon (Skrzycki and Czeczot, 2013). Similar results were observed in experiments carried out *in vitro* and *in vivo* (rodent models), where Sigmar1 was essential for the growth of prostate cancer, breast cancer, and colorectal cancer cells. Upregulated levels of Sigmar1 in different cancer cell lines drive cell migration, invasiveness, and promote cell survival by increasing calcium entry in the cells and regulating membrane electrical activity (Crottes et al., 2016; Gueguinou et al., 2017). Inhibition of Sigmar1 by ligands limited the translocation of androgen receptor and mediated protective effects in prostate cancer cells (Thomas et al., 2017). Additionally, Sigmar1 inhibition altered calcium homeostasis increased apoptotic cell death, and inhibit cancer cell proliferation and migration in breast cancer and colorectal cancer cells (Azzariti et al., 2006; Crottes et al., 2016; Gueguinou et al., 2017). In contrast, hepatocarcinoma cells have been shown to have reduced Sigmar1 expression in clinical samples and human liver cancer cells (HepG2) concurrent with reduced apoptosis and increased NF-κB levels. Overexpression of Sigmar1 *in vitro* in HepG2 cells has demonstrated protective effects by reducing cell proliferation, increasing apoptosis, and decreasing NF-κB levels (Xu et al., 2018). However, inhibition of Sigmar1 in hepatocarcinoma also showed protective effects through the reduction of iron metabolism and ferroptosis (Bai et al., 2019). The discrepancy between study results may be related to the focused signaling pathway (ferroptosis vs. apoptosis, cell proliferation vs. mitochondrial ROS).

All these studies demonstrate a potential regulatory role for Sigmar1 in cancer biology, as Sigmar1 has been shown to be upregulated in all types of cancer and functions in driving cell migration, increasing membrane invasiveness, and further enhancing cell proliferation, contributing to disease progression. However, further studies are required to understand the exact mechanism and functions of Sigmar1 in different cancer cells.

### Retinal Diseases

Sigmar1 expression in the various ocular tissues was first reported using pharmacological ligand binding assays (Senda et al., 1998) and biochemical experiments (Ola et al., 2001, 2002). Subsequent studies have shown Sigmar1 expression in multiple retinal cell types, including photoreceptor cells, ganglion cells, and Müller and pigment epithelial cells (Ola et al., 2002; Jiang et al., 2006). Sigmar1 null mice demonstrate normal retina development, but as they aged, these mice developed apoptosis in the optic nerve head, decreased ganglion cell function, and eventually loss of ganglion cells (Ha et al., 2011). Extensive research on the molecular function of Sigmar1 showed pharmacologic Sigmar1 activation by agonists attenuated ganglion cell death (Campana et al., 2002; Smith et al., 2008), mitigated retinal glial cell reactivity (Zhao et al., 2014; Wang et al., 2015; Vogler et al., 2016), and diminished light-induced photoreceptor cell loss (Shimazawa et al., 2015). Sigmar1’s role in diabetic retinopathy was evident from two murine models of diabetic retinopathy, the streptozotocin-induced model (Ola et al., 2002) and the Ins2Akita/+ mouse (Smith et al., 2008). Both of these diabetic models showed a similar level of Sigmar1 expression in the retinal tissues compared to control mice (Ola et al., 2002; Smith et al., 2008). Pharmacologic activation of Sigmar1 in the *Ins*2^Akita/+^ mouse conferred significant neuroprotection, reduced oxidative stress, and preserved retinal architecture (Smith et al., 2008). The protective effect of Sigmar1 in diabetes-induced retinal neurodegeneration has also been demonstrated using Sigmar1 null mice, where the absence of Sigmar1 aggravated retinal ganglionic cell dysfunction in streptozotocin-injected diabetic mice (Ha et al., 2012). Sigmar1 activation by treatment with agonists also showed neuroprotective effects associated with attenuated Müller cell gliosis, reduced microglial activation, and decreased oxidative stress in an inherited photoreceptor degeneration model, the Pde6brd10/J (rd10) mouse model (Wang et al., 2016). Extensive *in vivo* and *in vitro* mechanistic studies have been performed in isolated retinal cells, such as Müller glial cells, microglial cells, optic nerve head astrocytes, and retinal ganglion cells as well as in the intact retina to determine the molecular signaling pathways regulated by Sigmar1 (Smith et al., 2018). All these studies together demonstrate that Sigmar1 dependent retinal neuroprotection involved activation of functions associated with ion channel regulation, chaperone activity, oxidative stress modulation, and regulation of cellular calcium (Smith et al., 2018).

### Kidney Injury

Expression of Sigmar1 has also been detected in kidneys using Northern blot and Western blot analysis, suggesting a potential role for Sigmar1 in kidney pathophysiology. Sigmar1 expression level were found to be significantly reduced during kidney injury in rats induced by pressure overload following bilateral ovariectomy (Bhuiyan and Fukunaga, 2010). Sigmar1 activation following dehydroepiandrosterone treatment was found to prevent kidney injury by activating the Akt-eNOS signaling pathways and restoring NO levels (Bhuiyan and Fukunaga, 2010). Similarly, Sigmar1 agonists (Fluvoxamine) have been shown to improve postischemic survival and renal function via activation of Akt-mediated nitric oxide signaling in the kidney in rats model of ischemia-reperfusion injury (Hosszu et al., 2017). A recent study has provided evidence for increased Sigmar1 expression in distal tubular kidney cells of young and streptozotocin (STZ) induced diabetic rats (Milardovic et al., 2020). However, the molecular role for Sigmar1 in the postnatal development of the rat kidneys and in distal tubular damage in the pathogenesis of diabetes requires further investigation.

### COVID-19

Sigma ligands has recently been explored as a therapeutic target in COVID-19 repurposing therapy (Abate et al., 2020; Vela, 2020; Hashimoto, 2021). Both Sigmar1 agonist (fluvoxamine) (Lenze et al., 2020) and antagonist (haloperidol) (Hoertel et al., 2021) underwent clinical trial for possible therapy in patients with COVID-19. The interaction map for SARS-CoV-2 protein reveals Sigmar1 interaction with Nsp6 (SARS-CoV-2 viral protein) and proposed Sigmar1 ligands as a possible therapeutic target for COVID-19 (Gordon et al., 2020b). Validation of the interaction map showed Sigma ligands (both Sigmar1 and Sigmar2) inhibit viral activity (Gordon et al., 2020b). Further studies of the role of Sigmar1 in COVID-19 have suggested a functional host-dependency factor for SARS-CoV-2; the absence of Sigmar1 by knockdown reduced the replication of SARS-CoV-2 protein, delaying disease progression and presenting Sigmar1 as an attractive therapeutic target (Gordon et al., 2020a). Extensive future research are required to elucidate the molecular role of Sigmar1 in COVID-19 pathobiology.

### Others

**FIGURE 4:**
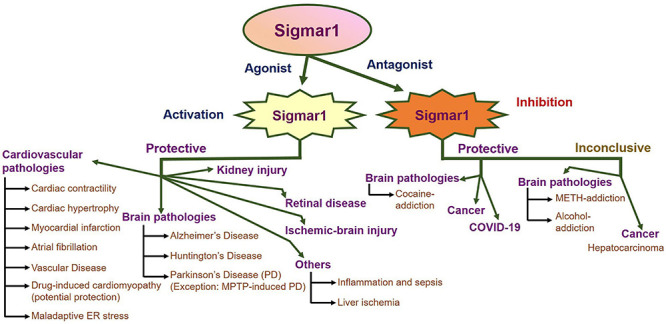
Summary of the pathophysiological functions of Sigmar1. Schematic showing an overall summary of the role of Sigmar1 in the pathophysiology related to different organs as present in the current literature. Briefly, agonist-mediated activation of Sigmar1 has protective effects in pathological conditions of several organs including heart (cardiac hypertrophy, myocardial infarction, atrial fibrillation, vascular disease, drug-induced cardiomyopathy and maladaptive ER stress), brain (neurodegenerative diseases including AD, HD and PD (with the exception of MPTP-induced PD) and ischemic brain injuries), kidneys, retina, liver, and the immune system. Inhibition of Sigmar1 using its antagonists is reported to be protective in several pathologies including cancer, cocaine addiction, and COVID-19. However, due to conflicting reports on whether activation or inhibition of Sigmar1 is protective, the field remains inconclusive about the effects of Sigmar1 on methamphetamine and alcohol addiction.

In addition to the association of Sigmar1 with the above mentioned pathologies (summarized in Figure 4), the protective role of Sigmar1 has also been explored in inflammation and sepsis. During sepsis, Sigmar1 has been shown to interact with IRE1, reducing the splicing of XBP1, resulting in reduced production of inflammatory cytokines and increasing the longevity of mice in sub-lethal models of sepsis (Rosen et al., 2019). These protective effects of Sigmar1 were confirmed using Sigmar1 null mice, where the absence of Sigmar1 further increased pro-inflammatory cytokines, XBP1 splicing, and reduced survival of mice (Rosen et al., 2019). Sigmar1 has also been implicated in liver ischemia where the use of Sigmar1 ligand BHDP (a sigmar1 ligand) has been shown to maintain membrane integrity, restore metabolic capacities of the liver, restore mitochondrial respiration and tissue integrity, reducing the deleterious effects caused by ischemia in the liver (Klouz et al., 2008). All these studies suggest strong involvement of Sigmar1 in different pathologies, and more studies are required to further explore it as a therapeutic target.

## Biological Functions of Sigmar1

**FIGURE 5:**
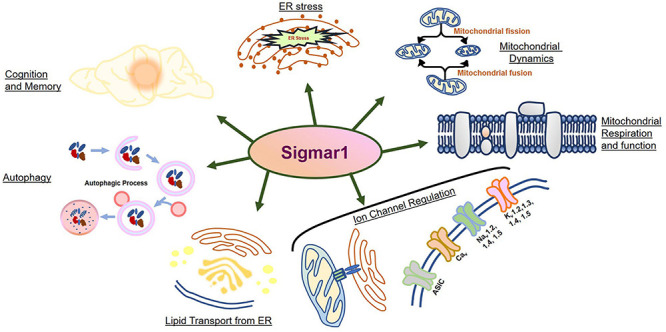
Biological functions of Sigmar1. Schematic diagram summarizing the functions of Sigmar1 including its involvement in cognition, memory, ER stress, mitochondrial dynamics, mitochondrial respiration and function, autophagy, lipid transport from ER, and ion channel regulation.

Despite extensive research on Sigmar1 over the last fifty years, we are still exploring and learning about the molecular role of Sigmar1 in different cells and organs. Research to date suggested that Sigmar1 mediates cellular signaling pathways by acting as a ligand-operated chaperone protein, and lack its intrinsic signaling machinery (Figure 5). Sigmar1 functions primarily via translocation and protein-protein interactions by ligands to modulate the activity of various ion channels and signaling molecules, including inositol phosphates, protein kinases, and calcium channels (Su et al., 2010). Most of these studies were performed in cell culture models using Sigmar1 ligands (agonist or antagonists), and most of these ligands possess affinities for other receptors or even elicit pleiotropic effects. Moreover, the molecular characteristics of Sigmar1’s interactions in each of these signaling pathways remain elusive.

### Ion Channel Regulation

Earlier studies on Sigmar1 have suggested that Sigmar1 dependent modulation of ion channel signaling pathways occurs via protein-protein interaction as summarized in Table 3. Extensive research has suggested that Sigmar1 may be involved in intracellular calcium signaling and inositol triphosphate (IP3) turnover as evident by Sigmar1 interaction with Inositol triphosphate receptors (ITPR) using co-immunoprecipitation (Co-IP). Sigmar1 interaction with all three types of ITPRs (ITPR1, ITPR2, and ITPR3) has been reported using *in vitro* experiments in different cells, and ligand-dependent activation of Sigmar1-ITPR interaction modulates the intracellular calcium levels (Hayashi and Su, 2007). Sigmar1 was reported to form a trimeric complex with ITPR3 and Ankyrin B regulating ER-mitochondrial communication through regulating Ca2+ efflux from the ER into the cytosol (Hayashi and Su, 2001; Wu and Bowen, 2008). Sigmar1 inhibition by antagonists also showed to decrease in calcium response in neuroblastoma cells (Gasparre et al., 2012). In hepatocytes, Sigmar1 stimulation decreased IP3R1 dependent calcium signaling by inhibiting the synthesis of IP3 receptors in PKC dependent manner (Abou-lovergne et al., 2011). In cardiomyocytes, Sigmar1 stimulation reduced upregulation of pressure-overload induced IP3R-2 (Tagashira et al., 2013a). This study further reported binding of Sigmar1 to ryanodine receptor (RyR), and Sigmar1 activation by ligands showed inhibition of ryanodine-induced calcium release from the sarcoplasmic reticulum (SR) in cardiomyocytes (Tagashira et al., 2013a). All these studies point toward a cell-type dependent effect of Sigmar1 stimulation by agonist on ITPR dependent calcium release.

**TABLE 3:** Sigmar1 dependent regulation of ion channels through protein-protein interaction.

**Ion channel**	**Cell types**	**Method of interaction detection**	**Sigmar1 construct**	**Ion Channel construct**	**Expression**	**References**
Inositol tri-phosphate receptor (IP3R)	NG-108, CHO, bovine brain mitochondria	Co-IP	Native Sigmar1 and Sigmar1-EGFP	Native IP3R3	Native expression and transient expression	Hayashi and Su, 2001, 2007; Natsvlishvili et al., 2015
	NG-108	Co-IP and PLA	Native Sigmar1	Native IP3R1	Native expression	Kubickova et al., 2018
	Rat heart tissue	Co-IP	Native Sigmar1	Native IP3R2	Native expression	Tagashira et al., 2013a
Ryanodine receptor	Rat heart tissue	Co-IP	Native Sigmar1	Native RYR2	Native expression	Tagashira et al., 2013a
L-ype Voltage-gated calcium channel (VGCC)	RGC-5 cells	Co-IP	Wild-type Sigmar1	Native L-type VGCC	Stable and native expression	Tchedre et al., 2008
Voltage-dependent N-type calcium channel (Ca_v_2.2)	HEK293T	FRET and Co-IP	Sigmar1-dsred	EGFP-Ca_v_2.2	Transient overexpression	Zhang et al., 2017c
Calcium release-activated calcium channel	tSA-201	Co-IP	Sigmar1-FLAG	ORAI-Myc	Transient overexpression	Srivats et al., 2016
Voltage-dependent potassium channel	Mouse nucleus accumbens lysate, NG108-15	Co-IP and Co-IP with cross-linking	Native Sigmar1 and Sigmar1-V5-His	Native K_v_1.2, wild-type K_v_1.2	Native expression and transient expression	Kourrich et al., 2013
	HEK293	Co-IP	Sigmar1-FLAG	K_v_1.3-HA	Transient expression	Kinoshita et al., 2012
	Rat posterior pituitary lysate	Co-IP	Native Sigmar1	Native K_v_1.4	Native expression	Aydar et al., 2002
SK3 channel	SKmel28 cells, HEK293	Co-IP and HTRF	Sigmar1-Myc, HALO-Sigmar1-Myc	Wild-type SK3, SK3-HA	Transient and stable overexpression	Gueguinou et al., 2017
Na_v_1.5 Na^+^ channel	tSA201, MDA-MB-468, MDA-MB-231	Anti-FLAG chromatography, PLA, Co-IP	Sigmar1-FLAG, Native Sigmar1	Na_v_1.5-HA, Native Na_v_1.5	Transient overexpression, native expression	Balasuriya et al., 2012
Voltage-dependent anion channel 2 (VDAC2)	MA-10	Co-IP	Native Sigmar1	Native VDAC2	Native expression	Marriott et al., 2012
Acid sensing ion channel 1a (ASIC1a)	HEK293	Ni affinity chromatography	Sigmar1-FLAG-His	ASIC1a-His	Stable overexpression (ASIC1a) and transient overexpression (Sigmar1)	Carnally et al., 2010

*Co-IP, Co-immunoprecipitation; PLA, proximity ligation assay; FRET, fluorescence resonance energy transfer; HTRF, homogeneous time resolved fluorescence.*

Apart from the ITPRs, Sigma ligands have also been shown to block all calcium channel subtypes (N-, L-, P/Q-, and R-type calcium channels) in neonatal rat intracardiac and superior cervical (SCG) ganglia (Zhang and Cuevas, 2002). Several studies showed that the Sigmar1 ligand could modulate calcium influx in the cells by regulating voltage-dependent calcium channel (VDCC) (Hayashi et al., 2000). Use of Sigmar1 ligands inhibited calcium re-entry resulting in decreased calcium current in the cells (Katnik et al., 2006; Amer et al., 2013; Pan et al., 2014). This antagonism by Sigmar1 on VDCC and calcium current were used to treat painful neuropathies, reduce the unwanted increase in vascular permeability and angiogenesis, target excitotoxicity-induced neurodegenerative disease, and confer neuroprotection (Katnik et al., 2006; Amer et al., 2013; Pan et al., 2014). Another mechanism of Sigmar1 dependent regulation of Ca^2+^ dynamics has been demonstrated through store-operated calcium entry by modulating Orai to STIM1 coupling (Brailoiu et al., 2015; Srivats et al., 2016). Studies have shown that Sigmar1 activation by agonists induced Sigmar1-STIM1 interactions and de-coupled it from the Orai complex, and thereby inhibiting calcium influx (Brailoiu et al., 2015; Srivats et al., 2016).

Several Sigmar1 ligands have been shown to regulate the activity of a different type of potassium channels (Kv1.2, Kv1.3, Kv1.4, and Kv1.5) in different cell types (NG108, and oocytes) (Mavlyutov et al., 2010; Kinoshita et al., 2012; Kourrich et al., 2013). Activation of Sigmar1 by agonists decreased the transient outward potassium current (Soriani et al., 1999a) and the sustained outward potassium current (Soriani et al., 1999b) in cultured frog pituitary melanotrope cells. Sigmar1 activation also inhibited Kv1.4 potassium channels in Xenopus oocytes by interaction with Kv1.4 (Aydar et al., 2002). Sigmar1 activation by ligands also reversibly blocked delayed outwardly rectifying potassium channels, large-conductance Ca^2+^-sensitive K^+^ channels, and the M-current by direct coupling to potassium channels in rat parasympathetic intracardiac neurons (Zhang and Cuevas, 2005). In addition, Sigmar1 has also been shown to bind to a voltage-dependent sodium channel (Nav1.5) and Sigmar1 ligands (haloperidol and pentazocine) altered Sigmar1: sodium channel interaction (Balasuriya et al., 2012; Aydar et al., 2016). Activation of Sigmar1 with agonists reduced voltage-gated Na+ channel (namely Nav1.5, Nav1.2/1.4) function in neonatal rat intracardiac ganglia, cardiomyocytes, and HEK-293 cells. These resulted in alterations in the action potential configuration with increased latency and reduced firing (Johannessen et al., 2009; Zhang et al., 2009; Gao et al., 2012). Sigmar1 was also reported to modulate acid-sensing ion channels (ASIC) (specifically, ASIC1a, a channel with higher permeability for ca2+ ions) through direct interaction (Herrera et al., 2008; Carnally et al., 2010). Activation of Sigmar1 using agonists inhibited ASIC1a function, decreased membrane currents, reduced acidosis induced ion flux, and increased calcium-induced current ([Ca2+]i) (Herrera et al., 2008).

Despite extensive studies that support the modulation of ion channel signaling by Sigmar1 ligands, more research is required to determine the direct involvement of Sigmar1 through Sigmar1-protein interactions. Studies have shown that several Sigmar1 ligands directly modulate ion channel function independently of the Sigmar1 (Gao et al., 2012; Amer et al., 2013; Brindley et al., 2017; Asano et al., 2019). Two Sigmar1 ligands (BD1047/BD1063 and 4−IBP) have been shown to inhibit chemically activated calcium entry channels [transient receptor potential (TRP)], acting relatively directly and independently of the Sigmar1 in human saphenous vein endothelial cells and HEK-293 cells (Amer et al., 2013). (+)-SKF 10047 inhibited NaV1.2 and NaV1.4 channels independently of sigma-1 receptor activation in HEK-293T cells and COS-7 cells (Gao et al., 2012). Similarly, some of the Sigmar1 ligands used have a very weak affinity for Sigmar1, requiring high concentrations (>10 μM) of the Sigmar1 ligands to allow observation of ion channel activity (Hayashi et al., 2000; Zhang and Cuevas, 2002; Johannessen et al., 2009; Zhang et al., 2017b). Overall, more research is required to understand the molecular mechanism responsible for Sigmar1 dependent modulation of ion channels.

### Chaperone Function

It has been proposed that Sigmar1 functions as a ligand-operated chaperone following identifications of a large number of protein-protein interactions between Sigmar1 and other proteins. The chaperone activity shown by Sigmar1 has been demonstrated by using reconstituted biochemical experiments where a purified C-terminal fragment of the Sigmar1 (residues 116-223) minimized the aggregation of proteins in a light scattering assay (Ortega-Roldan et al., 2013; Gregianin et al., 2016). Additional indirect studies have also shown evidence for Sigmar1’s chaperone function as Sigmar1 overexpression increased the whole-cell or surface expression of various proteins (Crottes et al., 2011; Kourrich et al., 2013; Pabba et al., 2014). Similarly, Sigmar1 knockdown *in vitro* also decreased the expression of several proteins suggesting chaperone function (Hayashi and Su, 2007; Mori et al., 2013; Aydar et al., 2016). The increased stability of different proteins, including IP3R channels (at the ER-mitochondria interface) (Gregianin et al., 2016), ankyrin (Hayashi and Su, 2001), potassium channels (Aydar et al., 2002), opioid receptors (Kim et al., 2010; Hong et al., 2017), and dopamine receptors (D1 and D2) (Kim et al., 2010; Navarro et al., 2010, 2013), following ligand-dependent activation of Sigmar1 also supports Sigmar1’s chaperone activity. Additionally, it has been proposed that the chaperone function of Sigmar1 is part of ER-associated degradation (ERAD) machinery involving in degradation of sphingolipid enzymes (Hayashi et al., 2012). Studies have also reported that Sigmar1 modulates the ER stress response and subsequent unfolded protein response (UPR), influencing protein stability and localization (Mori et al., 2013; Rosen et al., 2019). In addition, Sigmar1’s chaperone activity has been reported to degrade intranuclear inclusions and provide neuroprotection in Huntington’s disease, Alzheimer’s disease, and Parkinson’s disease (Miki et al., 2014, 2015; Yamoah et al., 2020). More studies are required to demonstrate the molecular mechanism responsible for the chaperone function of Sigmar1 *in vivo*.

### Regulation of Mitochondrial Morphology, Dynamics, and Function

Evidence for the molecular role played by Sigmar1 in mitochondrial morphology, dynamics, and function comes from studies involving the Sigmar1 null mouse and phenotypes of the disease-causing mutations found in Sigmar1. The involvement of Sigmar1 in mitochondrial morphology has been made evident by studies where the loss of Sigmar1, either by knockdown or by pharmacologic inactivation with the antagonist (using NE-100) resulted in increased mitochondrial length and development of mitochondrial dysfunction (Bernard-Marissal et al., 2015). In contrast to the existing notion stating Sigmar1 inhibition results in mitochondrial elongation, one study showed overexpression of full length and a spliced variant of Sigmar1 (with a deletion of 47 base pairs starting at amino acid 106 further resulting in a shorter form of Sigmar1) increased mitochondrial length (Shioda et al., 2012). This study involved *in vitro* experiments using Sigmar1 overexpressed (full length and spliced Sigmar1) Neuro-2a cells. However, further studies are required to demonstrate the role of Sigmar1 in regulating mitochondrial dynamics and define the molecular mechanisms thereof.

The molecular role played by Sigmar1 in regulating mitochondrial calcium signaling has been reported by studies showing that Sigmar1 interacts with IP3R3 to regulate ER-mitochondrial calcium levels under ER-stress conditions in CHO and neuroblastoma cells (Hayashi and Su, 2007). Expression of the mutant SIGMAR1 resulted in a non-functional Sigmar1 caused by mislocalization from MAM, impairing Sigmar1-IP3R3 interaction and thereby altering intracellular and mitochondrial calcium handling as in human neuroblastoma cell lines (SH-SY5Y and SK-N-BE), murine motor neuron-like NSC-34 cells, and N2a cells (Gregianin et al., 2016; Watanabe et al., 2016). Similarly, the absence of Sigmar1 in motor neuron cells from Sigmar1 null mice and inactivation by antagonist NE-100 (in motor neuron cells) showed impairment of ER-mitochondria contacts, deregulation of calcium homeostasis, and activation of ER stress pathways. Sigmar1 activation by agonist (PRE-084) showed protective effects in these cell lines restoring the Sigmar1-IP3R3 interaction and preserving the calcium homeostasis (Bernard-Marissal et al., 2015; Watanabe et al., 2016). Moreover, overexpression of mutant Sigmar1 in cells resulted in collapsing of mitochondrial-associated ER membrane leading to deregulated calcium signaling (Dussossoy et al., 1999; Gregianin et al., 2016). Similarly, Sigmar1 mutant overexpression in neuro2A cells resulted in mitochondrial dysfunction and reduced ATP production. Supplementation with methyl pyruvate (TCA cycle substrate) enhanced ATP production and rescued the mitochondrial dysfunction (Fukunaga et al., 2015). Moreover, Sigmar1 has been shown to regulate mitochondrial metabolism where Sigmar1 drives cholesterol influx to the mitochondria through interaction with VDAC2 (Marriott et al., 2012).

Expression of a spliced variant of Sigmar1 results in a shorter version of the Sigmar1 protein and subsequently reduced ATP production, increase ER stress, increase autophagosome formation, and increased apoptosis in Neuro2a cells (Shioda et al., 2012). Additionally, Sigmar1 has been shown to be associated with mitochondrial metabolic regulation regulating conversion of cholesterol to pregnenolone and mediate steroidogenesis in MA-10 cells (Leydig tumor cells) (Marriott et al., 2012). Knockdown of Sigmar1 by siRNA in MA-10 cells reduced pregnenolone synthesis by more than 75% (Marriott et al., 2012). Pharmacologic activation of Sigmar1 by agonists enhances mitochondrial complex I activity and increased mitochondrial ROS production at the physiological condition in the forebrain of mice (Goguadze et al., 2019). However, under pathological conditions, i.e., in a mouse model of Alzheimer disease (mice expressing the Aβ1-42 and, Aβ25–35 peptides), activation of Sigmar1 has been shown to reduce mitochondrial ROS production (Lahmy et al., 2015; Goguadze et al., 2019). Further studies have suggested that activation of Sigmar1 using agonists provided protection against Aβ25–35 peptide induced reduction in oxygen consumption during all states of mitochondrial respiration, mitochondrial complex IV activity, and mitochondrial damage (as assessed by increased cytochrome C release) (Lahmy et al., 2015).

Sigmar1’s molecular role in the regulation of mitochondrial function has also been reported in the retinal cells. Sigmar1 overexpression and pharmacologic activation protected and restored mitochondrial membrane potential and cytochrome C release in retinal ganglion cells isolated from rat pups exposed to hypoxia (Ellis et al., 2017). Additionally, the absence of Sigmar1 in retinal explants in Sigmar1 null mice and neuronal cell line showed reduced mitochondrial clearance upon mitophagy induction without the involvement of PINK/Parkin mitophagy pathway (Yang et al., 2019). The converse was also true when using dopaminergic neurons where Sigmar1 activation by agonists (PRE-084) rescued the defects in mitophagy clearance in Parkinson’s disease in a PINK/Parkin dependent pathway (Wang et al., 2020). The involvement of Sigmar1 in neuroprotection was confirmed when dopaminergic neuronal cells isolated from Sigmar1 null mice showed impaired mitochondrial clearance with reduced levels of PINK and Parkin during mitophagy induction (Wang et al., 2020).

Similar effects have been observed in cardiomyocytes where the activation of Sigmar1 by agonists (SA4503, fluvoxamine) restored mitochondrial calcium mobilization and ATP production in angiotensin-induced cardiomyocyte hypertrophy (Tagashira et al., 2013c, 2014a). Similarly, pressure-overload induced hypertrophy in mice showed impaired calcium uptake and reduced ATP production, which were restored upon treatment with Sigmar1 agonists (SA4503, fluvoxamine) (Tagashira et al., 2013c, 2014a). These results were supported by experiments using Sigmar1 antagonists (NE-100, haloperidol), which demonstrated aggravated cardiac pathology in transverse aortic constriction (TAC)-subjected mice and angiotensin treated in cardiomyocytes (Tagashira et al., 2013c; Shinoda et al., 2016).

All these studies together suggest a molecular role for Sigmar1 in mitochondrial dynamics and functions in different cell types (including neuronal, retinal, and cardiac systems), modulation of mitochondrial calcium mobilization, mitochondrial ATP production, and mitochondrial lipid metabolism. However, the molecular mechanism responsible for Sigmar1 dependent regulation of mitochondrial dynamics and functions still remains unknown.

### Sigmar1 in Autophagy

Sigmar1’s molecular role in the autophagy pathway has been made evident by studies where Sigmar1 siRNA knockdown in HEK293 and NSC34 cells led to the accumulation of numerous autophagic vacuoles often filled with non-degraded autophagic substrates and deformities of ER ultrastructure (Prause et al., 2013; Vollrath et al., 2014). Subsequent studies using NIH-3T3 cells stably expressing RFP-GFP-LC3 showed that Sigmar1 siRNA knockdown impaired the fusion of endosomes or autophagosomes to lysosomes (Vollrath et al., 2014). TEM images also showed the accumulation of several double-membrane autophagosomes (AV) filled with cargos that failed to fuse with lysosomes in Sigmar1-deficient GFP–RFP-LC3 expressing NIH-3T3, NSC34, and HEK-293 cell lines (Vollrath et al., 2014). All these biochemical analysis suggested an impairment of endolysosomal pathways in Sigmar1-deficient cells indicated by the accumulation of various autophagic substrates and defects in endosomal trafficking.

In addition, Sigmar1 has been identified as a novel regulator of autophagosome expansion during starvation in a siRNA screen in RPE1 cells (MacVicar et al., 2015). The effect of Sigmar1 on autophagy has also been reported by studies showing Sigmar1 ligands modulated the autophagic process in a dose-dependent manner uveal melanoma cells (Schrock et al., 2013). Moreover, studies using cancer cell lines (e.g., breast cancer, hepato-carcinoma, pancreatic adenocarcinoma, prostate adenocarcinoma) have also shown Sigmar1 mediated regulation of autophagy where Sigmar1 inhibition by antagonists (IPAG [1-(4-iodophenyl)-3-(2-adamantyl) guanidine], haloperidol) increased autophagosome formation. Surprisingly, Sigmar1 activation by agonists [PRE-084, (+) SKF10047] in these cell lines did not show any effect onautophagy (Maher et al., 2018; Yang et al., 2019). Contrary to the results obtained from studies in cancer cells, knockout of Sigmar1 impaired mitochondrial clearance without altering the PINK1/Parkin signaling in mouse retinal explants and cultured cells (HEK-293, NSC34, and SH-SY5Ycell lines) (Yang et al., 2019). The study further showed that the absence of Sigmar1 partially impaired autophagosome and lysosome fusion in SNARE-dependent mechanism, with no effects on autophagosome closure or lysosome functional activity (Yang et al., 2019). Recently, a Sigmar1 agonist [tetrahydro-N,N-dimethyl-2,2-diphenyl-3-furanmethanamine hydrochloride (ANAVEX2-73)] has been shown to increase autophagy through ULK1 phosphorylation and to reduce proteotoxicity by decreasing protein aggregation in HeLa, HEK 293A cells, and *C. elegans* (Christ et al., 2019).

Despite the contradictory findings concerning the role of Sigmar1 in autophagy made by different groups (which may be due to the use of different cell types and the systems used under the study conditions), Sigmar1 has been showed to have a substantial role in the modulation of autophagy in maintaining cellular homeostasis. However, since all these studies were limited to *in vitro* cell culture models, which lacked proper monitoring of autophagy flux, the molecular mechanism responsible for Sigmar1 dependent regulations of autophagy (either activation or inhibition) *in vivo* remained elusive. Further studies are required to dissect the types of autophagy (macro-autophagy vs. mitophagy) regulated by Sigmar1, determine molecular mechanism responsible for Sigmar1-dependent regulation of macro-autophagy/mitophagy, and assess the effects of acute and chronic Sigmar1 dependent regulation of macro-autophagy/mitophagy under conditions of cellular pathobiology.

### Sigmar1 in Lipid Metabolism

Sigmar1’s molecular function in lipid metabolism has been suggested by several studies. Sigmar1 is specifically targeted to lipid storage sites (lipid droplets) (Hayashi and Su, 2003b) and regulates the compartmentalization of ER-synthesized neutral lipids (triglycerides and cholesteryl esters) in NG 108 cells (Hayashi and Su, 2003a, b; Hayashi and Fujimoto, 2010). Sigmar1 also accumulates at lipids rafts by forming a complex with cholesterol and galactosylceramides (GalCer) regulating the GalCer (Hayashi and Su, 2004) and regulates cholesterol transport in NG108 cells (Hayashi and Su, 2005). Sigmar1 also causes the remodeling of lipid rafts by increasing the level of lipid raft-forming gangliosides in PC12 cells (Takebayashi et al., 2004). All these studies were correlative, limited to *in vitro* experiments in cell lines [NG-108 cells (Hayashi and Su, 2003a, b)] and pharmacologic approaches using less selective ligands [such as (+)-pentazocine (Hayashi and Su, 2003a, b)]. Despite the preponderance of evidence provided by these studies, the molecular mechanisms of Sigmar1-dependent regulation of lipid metabolism remain unknown.

### Sigmar1 in ER Stress Response

The role of Sigmar1, as well as that of Sigmar1 ligands (agonists and antagonists), has been extensively studied under ER stress conditions in different cell systems and demonstrated a wide range of cellular effects depending on the cell type. The Sigmar1 agonist [(+)-Pentazocine] suppressed oxidative stress-induced cell death and suppressed the induction of the ER stress proteins BiP and EIF2α in the human lens cell line (Wang et al., 2012). In contrast, the Sigmar1 antagonist (NE-100) protected against the ER stress-induced cell death in murine hippocampal HT22 cells via CHOP expression by the upregulation of GRP78 through the ATF6 pathway, and these protective effects were independent of Sigmar1 antagonistic effect (Ono et al., 2013). However, Sigmar1 antagonist (NE-100) did not change the expression of phosphorylated eukaryotic initiation factor 2α (p-eIF2α) and splicing of X-box-binding protein 1 (XBP-1) in HT22 cells (Ono et al., 2013). Another study showed imipramine treatment in HT22 cell inhibited tunicamycin-induced cell death, which was abolished by treatment with NE-100 (Ono et al., 2012). ER-stress (induced by tunicamycin or thapsigargin) transcriptionally increased Sigmar1 protein levels via the PERK/eIF2α/ATF4 pathway and ameliorated cell death signaling under in HEK293 cells or mouse neuroblastoma (Neuro2a) cells (Mitsuda et al., 2011). In contrast, Sigmar1 agonist (fluvoxamine) induced Sigmar1 level by increasing ATF4 translation without the involvement of the whole PERK pathway in Neuro2a (Omi et al., 2014). Sigmar1 dependent regulation of the stress-inducible transcription factor, C/EBP-homologous protein (CHOP), was reported in primary cardiomyocytes in the tunicamycin-induced ER-stress model (Alam et al., 2017). Sigmar1-siRNA knockdown in neonatal rat ventricular cardiomyocytes (NRCs) could significantly increase the expression of CHOP and induced cellular toxicity by sustained activation of tunicamycin-induced ER stress. Conversely, adenovirus-mediated Sigmar1 overexpression decreased the expression of CHOP and significantly decreased cellular toxicity in cardiomyocytes. Mechanistically, Sigmar1-dependent activation of IRE1α-XBP1s ER-stress response pathways was associated with inhibition of CHOP expression and suppression of cellular toxicity in cardiomyocytes (Alam et al., 2017). All these studies together suggested that Sigmar1 functions as an essential component of the adaptive ER-stress response pathways but that the molecular ER-stress signaling and mechanisms varied depending on the ER-stress inducer and cell type. Therefore, more studies are required to explore the role of Sigmar1 in different pathophysiological conditions *in vivo* using Sigmar1 genetic mouse models.

### Perspective

Despite extensive studies carried out over the last fifty years, we are still at the beginning of our understanding of the molecular functions and cellular signaling mediated by Sigmar1. The majority of the studies to date have been limited to pharmacologic Sigmar1 ligands using *in vitro* cell culture models; data from *in vivo* genetic mouse models are needed to validate the cellular pathways mediated by these ligands. Extensive pharmacologic studies of the therapeutic role played by Sigmar1 in disease models has resulted in conflicting and confusing data due to the non-selectivity of the pharmacologic ligands, most of which possess affinity for other receptors or pleiotropic effects. To date the various Sigmar1 ligands tested to demonstrate the molecular role of Sigmar1 ranges from agonist, antagonists, reverse agonists, as well as some are Sigmar2 ligand (reviewed in Chu and Ruoho, 2016). Moreover, some of these ligands may also serve as a positive and negative allosteric modulators for Sigmar1 (reviewed in Vavers et al., 2019). The apparent complexity of these pharmacologic ligands requires elaborative review (Chu and Ruoho, 2016; Vavers et al., 2019). We presented a table of the most common ligands cited in this review article (Table 4).

**TABLE 4:** Most common Sigmar1 ligands cited in this manuscript with their respective affinities and selectivity.

**Ligand**	**Binding Affinity**	**Effect on Sigmar1**	**Effect on Sigmar2**	**Selectivity for others**	**Pleiotropic effects**
PRE-084	Sigmar1 (Ki 44–53 nM) > Sigmar2 (Ki 32.1 μM) (Su et al., 1991; Garcés-Ramírez et al., 2011)	Agonist (Maurice et al., 1994b; Hayashi and Su, 2007; Garcés-Ramírez et al., 2011; Hyrskyluoto et al., 2013; Gao et al., 2018)	−	Low binding affinity for dopamine D2, muscarinic acetylcholine, serotonin, and adrenergic receptors (Su et al., 1991)	−
SA4503 (cutamesine)	Sigmar1 (Ki 4.6 nM) > Sigmar2 (Ki 63.1 nM) (Matsuno et al., 1996)	Agonist (Kobayashi et al., 1996; Matsuno et al., 1996)	−	Little affinity for 36 other receptors, ion channels and second messenger systems (Matsuno et al., 1996)	−
(+) Pentazocine	Sigmar1 (Ki 3.9–23.3 nM) > Sigmar2 (Ki 1,542–6,611 nM) (Hellewell et al., 1994; John et al., 1999)	Agonist (Pan et al., 2014; Mishra et al., 2015)	Binds at low affinity (Hellewell and Bowen, 1990)	k-opioid receptor-partial agonist (analgesic), enhances acetylcholine release (Hiramatsu and Hoshino, 2005), low affinity partial agonist to mu receptor (Chien and Pasternak, 1994)	−
ANAVEX 2-73 (blarcamesine)	Moderate Sigmar1 (Ki 860 nM) (Villard et al., 2011)	Agonist (Vamvakidès, 2002; Villard et al., 2011; Christ et al., 2019)	−	Muscarinic acetylcholine receptor-inhibitor (Villard et al., 2011; Christ et al., 2019)	−
(+)-SKF-10047	Sigmar1 (Ki 54–597 nM) > Sigmar2 (Ki 11.17–39.74 μM) (Hellewell et al., 1994; Vilner and Bowen, 2000)	Agonist (Hellewell et al., 1994; Vilner and Bowen, 2000)	−	NMDA receptor antagonist, enhances acetylcholine release (Hiramatsu and Hoshino, 2005)	Inhibits Na_v_ channels in Sigmar1 knockout cells (Johannessen et al., 2009, 2011)
Fluvoxamine	Sigmar1 (Ki 36 nM) > Sigmar2 (Ki 8,439 nM) (Narita et al., 1996)	Agonist (Narita et al., 1996; Tagashira et al., 2010; Bhuiyan et al., 2011b)	−	Serotonin reuptake inhibitor (Fu et al., 2012; Narita et al., 1996)	−
DTG	Sigmar1 (Ki 38–203 nM) =Sigmar2 (Ki13–58 nM) (Koe et al., 1989; Hellewell et al., 1994; Vilner and Bowen, 2000)	Agonist (Walker et al., 1992; Marrazzo et al., 2011; Zampieri et al., 2016)	Agonist (Walker et al., 1992; Marrazzo et al., 2011; Zampieri et al., 2016)	−	Inhibits Na_v_ channels in Sigmar1 knockout cells (Johannessen et al., 2009, 2011)
DHEA	Moderate Sigmar1 (Ki 2.96 μM) (Maurice et al., 1996)	Agonist (Monnet et al., 1995; Waterhouse et al., 2007)	−	Neurosteroid, NMDA receptors, GABA-A receptors, nuclear receptor (Wu et al., 1991; Paul and Purdy, 1992; Waterhouse et al., 2007)	−
Cocaine	Sigmar1 (Ki 2.5–19 μM) > Sigmar2 (Ki 31 μM) (McCann and Su, 1991; Matsumoto et al., 2002; Garcés-Ramírez et al., 2011)	Agonist (Hayashi and Su, 2001, 2007; Matsumoto et al., 2002)	−	Dopamine transporters, Neurotransmitter reuptake blocker (Matsumoto et al., 2002; Beggiato et al., 2017)	−
4-IBP	Sigmar1 (Ki 1.70 nM) > Sigmar2 (Ki 25.2 nM) (John et al., 1995, 1998)	Agonist (Amer et al., 2013) Inverse agonist (Bermack and Debonnel, 2005; Schmidt et al., 2016)	Antagonist (John et al., 1995, 1998)	−	Inhibits Ca^2+^ entry and block TRPCs and TRPMs in the absence of Sigmar1 (Amer et al., 2013)
PPBP	Sigmar1 (Ki 0.8 nM) = Sigmar2 (Ki 1.14 nM) (Glennon et al., 1991; Hashimoto and London, 1993)	Agonist (Goyagi et al., 2001; Nishimura et al., 2008; Yang et al., 2010)	−	nNOS inhibitor (Goyagi et al., 2001; Yang et al., 2010)	−
Fabomotizole (Afobazole)	Moderate Sigmar1 (Ki 5.9 μM) (Seredenin and Voronin, 2009)	Agonist (Katnik et al., 2016)	Agonist (Katnik et al., 2016)	Melatonin receptors (MT_1_ and MT_3_), MAO-A receptive site (Kryzhanovskii et al., 2018)	−
**Pridopidine**	Sigmar1 (Ki 57 nM) > Sigmar2 (Ki 5,450 nM) (Johnston et al., 2019)	Agonist (Shenkman et al., 2021; Dyhring et al., 2010; Johnston et al., 2019)	Binds at comparatively lower affinity (Johnston et al., 2019)	adrenergic-α_2C_, Dopamine D2 and D3 receptors, serotoninergic-5HT_1A_ (Dyhring et al., 2010; Johnston et al., 2019)	−
Haloperidol	Sigmar1 (Ki 1–40 nM) Sigmar2 (Ki 12–221 nM) (Su et al., 1988: Hellewell et al., 1994; Vilner and Bowen, 2000; Choi et al., 2001)	Antagonist (Hellewell and Bowen, 1990; Hellewell et al., 1994; Mishra et al., 2015)	Antagonist (Hellewell and Bowen, 1990; Hellewell et al., 1994; Choi et al., 2001)	Dopamine (D2 and D3) receptor antagonist (Fox et al., 1994)	−
Sertraline	Sigmar1 (Ki 57 nM) > Sigmar2 (Ki 5,297 nM) (Narita et al., 1996)	Antagonist (Ishima et al., 2014) Inverse Agonist (Matsushima et al., 2019)	−	Serotonin reuptake inhibitor (Ishima et al., 2014)	−
PB28	Sigmar2 (Ki 0.28 nM) > Sigmar1 (Ki 10 nM) (Azzariti et al., 2006)	Antagonist (low affinity) (Azzariti et al., 2006)	Agonist (Azzariti et al., 2006)	−	Inhibits Na_v_ channels, and K_v_2.1 current in the absence of Sigmar1 (Johannessen et al., 2009, 2011; Liu et al., 2017)
NE-100	Sigmar1 (Ki 1.5 nM) > Sigmar2 (Ki 84.6 nM) (Okuyama et al., 1993)	Antagonist (Tanaka et al., 1995; Ono et al., 2013)		*In vivo* regulation of serotonin 5-HT2a receptors (Hashimoto et al., 1997)	Inhibits K_v_2.1 current in the absence of Sigmar1 (Liu et al., 2017)
E-5842	Sigmar1 (Ki 4 nM) > Sigmar2 (Ki 220 nM) (Guitart and Farré, 1998; Guitart et al., 1998; Romero et al., 2000)	Antagonist (Zamanillo et al., 2000)	−	Dopamine receptors, serotonin receptors, acetylcholine and muscarinic receptors, adrenergic receptors (Guitart and Farré, 1998)	Induces micronucleated polychromatic erythrocyte-dependent hypothermia in Sigmar1 null mice (Guzmán et al., 2008)
BD1047	Sigmar1 (Ki 0.6–5.3 nM) > Sigmar2 (Ki 47 nM) (Matsumoto et al., 1995)	Antagonist (Matsumoto et al., 1995; Pan et al., 2014)	Binds with 10 times lower affinity (Matsumoto et al., 1995)	Beta adrenergic receptor ligand (Matsumoto et al., 1995)	Inhibits Ca^2+^ entry and block TRP channels, and K_v_2.1 current in the absence of Sigmar1 (Amer et al., 2013; Liu et al., 2017)
BD1063	Sigmar1 (Ki 47 nM) > Sigmar2 (Ki 449 nM) (Matsumoto et al., 1995)	Antagonist (Matsumoto et al., 1995; Pan et al., 2014)	−	−	Inhibits Ca^2+^ entry and block TRP channels in the absence of Sigmar1 (Amer et al., 2013)
SM21	−	−	Antagonist (Ghelardini et al., 2000)	−	Inhibits K_v_2.1 current in the absence of Sigmar1 (Liu et al., 2017)
Methamphetamine	−	Antagonist (Hayashi and Su, 2007) Inverse agonist (Yasui and Su, 2016)	−	Dopamine Transporters, Monoamine transporters (Siciliano et al., 2014; McFadden et al., 2015)	−
IPAG	Sigmar1 (Ki 3 nM) > Sigmar2 (Ki 500–8000 nM) (Brimson et al., 2011; Schrock et al., 2013)	Antagonist (Schrock et al., 2013; Maher et al., 2018)	−	−	−

Overall, Sigmar1 may be involved in a wide range of vital cellular functions, including regulation of ion channel dynamics, modulation of protein stability via its chaperone activity, regulation of mitochondrial dynamics and function, and regulation of autophagy. This makes Sigmar1 an attractive through which to modulate the above mentioned cellular processes in different pathologies involving alteration or dysfunction of calcium homeostasis, protein aggregation, accumulation of dysfunctional mitochondria, and altered lipid content and metabolism. Though Sigmar1 is ubiquitously present in different organs, its molecular role and signaling mechanisms remain elusive in different cell types using genetic mouse models.

## Author Contributions

RA and MSB conceptualized, designed, and wrote the manuscript. RA, CSA, MM, and NSR participated in the conceptualization and editing of the manuscript. All co-authors edited and proofread the manuscript and approved the final version.

## Conflict of Interest

The authors declare that the research was conducted in the absence of any commercial or financial relationships that could be construed as a potential conflict of interest.

## Footnotes

1 https://www.proteinatlas.org

2 http://www.informatics.jax.org/external/ko/lexicon/2691.html
